# Locus-specific human endogenous retroviruses reveal lymphoma subtypes

**DOI:** 10.1016/j.isci.2025.112541

**Published:** 2025-04-28

**Authors:** Bhavya Singh, Nicholas Dopkins, Tongyi Fei, Jez L. Marston, Stephanie Michael, Helena Reyes-Gopar, Gislaine Curty, Jonas J. Heymann, Amy Chadburn, Peter Martin, Fabio E. Leal, Ethel Cesarman, Douglas F. Nixon, Matthew L. Bendall

**Affiliations:** 1 Division of Infectious Diseases, Department of Medicine, Weill Cornell Medicine, New York, NY 10021, USA; 2 Department of Immunology and Immunotherapy, Icahn School of Medicine at Mount Sinai, New York, NY 10029, USA; 3 Institute of Translational Research, Feinstein Institutes for Medical Research, Northwell Health, Manhasset, NY 11030, USA; 4 Department of Molecular Medicine, Zucker School of Medicine at Hofstra/Northwell-Hofstra University, Hempstead, NY 11549, USA; 5 Programa de Doctorado en Ciencias Biomédicas, Universidad Nacional Autónoma de México, Mexico City, Ciudad de México 04510, Mexico; 6 Departamento de Genómica Computacional, Instituto Nacional de Medicina Genómica, Mexico City, Ciudad de México 14610, Mexico; 7 Brazilian National Cancer Institute (INCA), Rio de Janeiro, Rio de Janeiro 20230-130, Brazil; 8 Department of Pathology and Laboratory Medicine, Weill Cornell Medicine, New York, NY 10021, USA; 9 Division of Hematology and Medical Oncology, Weill Cornell Medicine, New York, NY 10021, USA

**Keywords:** Genomics, Virology

## Abstract

The heterogeneity of cancers is driven by diverse mechanisms underlying oncogenesis such as differential ‘cell-of-origin’ progenitors, mutagenesis, and viral infections. Classification of B cell lymphomas has been defined by considering these characteristics. However, the expression and contribution of endogenous retroelements (EREs) to B cell lymphoma oncogenesis or classification have been overlooked. We hypothesized that incorporating ERE expression signatures would increase the resolution of B cell identity during healthy and malignant conditions. Here, we present the first comprehensive, locus-specific characterization of ERE expression in benign germinal center B cells, diffuse large B cell lymphoma, Epstein-Barr virus (EBV)-positive and EBV-negative Burkitt lymphoma, and follicular lymphoma. Our findings demonstrate unique human ERE signatures in the GC and lymphoma subtypes whose activity can be used in combination with gene expression to define B cell lineage in lymphoid malignancies, highlighting the potential of ERE analyses as a tool in lymphoma classification, diagnosis, and the identification of treatment groups.

## Highlights

• HERV expression distinguishes specific B cell subsets
• B cell lymphomas have distinct HERV signatures corresponding to their cell-of-origin
• Expression of HERVs in Burkitt lymphoma are associated with EBV status
• HERV-driven clustering of diffuse large B cell lymphoma is associated with survival

## Graphical abstract

**Figure:**
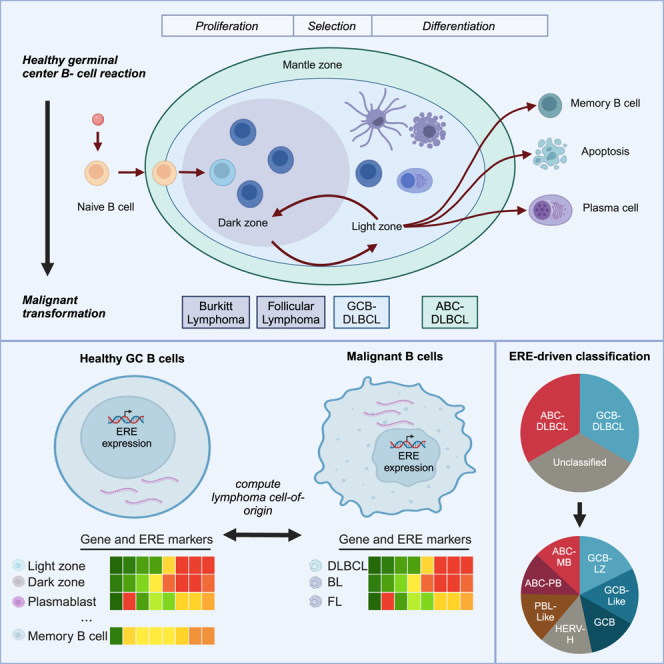

## Introduction

Endogenous retroelements (EREs) account for a substantial portion of the human genome.^1^^,^^2^ EREs include short interspersed nuclear elements (SINEs), long interspersed nuclear elements (LINEs), and human endogenous retroviruses (HERVs).^3^^,^^4^^,^^5^ HERVs are the remains of ancient retroviral infections that integrated within the germline.^6^^,^^7^ Since their integration, HERVs have accumulated mutations and deletions, but some of them have been co-opted by the host and can mediate key physiological processes.^8^^,^^9^^,^^10^^,^^11^^,^^12^^,^^13^^,^^14^ Under some conditions, the de-repression of HERVs can be associated with viral infectivity, pathogenic inflammation, and oncogenesis.^15^^,^^16^^,^^17^^,^^18^^,^^19^ Regulation of their expression is thought to be a driving factor in the initiation and sustainment of some human diseases.^20^^,^^21^^,^^22^

The germinal center (GC) is a focal component of the adaptive immunity, where naive B cells (NBs) travel to the follicles of secondary lymphoid organs to respond to T cell dependent antigen challenges.^23^ Through repeated cycling of proliferation and somatic hypermutation in the dark zone (DZ) and affinity selection in the light zone (LZ), B cells terminally differentiate into either memory B cells (MBs) or plasmablasts (PBs) in the GC.^23^ Following development in the GC, PBs then migrate to the bone marrow to facilitate long-term humoral immunity by becoming bone marrow plasma cells (BMPCs).^24^ The predicted “cells-of-origin” (COO) for certain non-Hodgkin B cell lymphomas are derived from B cells of the GC, as indicated by the detection of somatically mutated immunoglobulin genes.^25^ Burkitt lymphoma (BL) is thought to be derived from the DZ, while follicular lymphoma (FL) and germinal center B cell (GCB)-diffuse large B cell lymphomas (DBLCL) resemble LZ cells, and activated B cell like (ABC)-DLBCLs are broadly derived from GC cells arrested during plasma cell differentiation.^25^^,^^26^

In malignant transformation events, B cell development in the GC is expropriated and gives rise to lymphomagenic B cells.^25^ Oncogenic transposable elements (TE)-gene chimeric transcripts have been identified in a subset of DLBCL cases,^27^ and HERV dysregulation has been observed in response to EBV^28^^,^^29^ and human immunodeficiency virus-1 (HIV-1)^30^^,^^31^^,^^32^^,^^33^^,^^34^ infections, both of which are associated with BL and DLBCL. Transactivation of EREs by cancer-associated viruses such as with Epstein-Barr virus (EBV) could help drive the heterogeneous development of non-Hodgkin B cell lymphomas.^27^^,^^28^^,^^35^^,^^36^^,^^37^^,^^38^^,^^39^ In particular, EBV infection is known to alter the landscape of chromatin accessibility in infected B cells.^40^ B cells transformed by EBV infection exhibit higher transcription of long terminal repeats and several ERV families, along with overall DNA hypomethylation and increased transcription factor binding at ERV sites.^41^ Similarly, hepatitis B virus (HBV)-associated B cell lymphomas have a distinct transcriptional and mutational landscape.^42^^,^^43^ This heterogeneity in aggressive B cell lymphomas is also driven by other confounding factors, such as translocations events occurring at immunoglobulin, proto-oncogene, and tumor suppressor gene loci, somatic mutations, and often, differential COOs derived from the GC.^26^^,^^44^^,^^45^^,^^46^^,^^47^ B cell lymphomas have been subcategorized by classifiers such as LymphGen^47^ and EcoTyper^48^ to aid in treatment selections, however, these classifications have not considered ERE expression.

To further define these transformation events, we combined HERV signatures with gene expression data in GCB cells, DLBCL, EBV-positive and negative BL, and FL to define their B cell lineage. In doing this, we were able to confirm that HERV expression in these non-Hodgkin B cell lymphomas corresponded with their GC COO.

Our results provide a comprehensive, locus-specific atlas of ERE expression in the healthy GC and non-Hodgkin’s B cell lymphomas. These results identify additional prognostic categories that can be useful for the development of treatment strategies.

## Results

### ERE RNA expression in B cell lymphomas and germinal center B cells: HERVs distinguish specific B cell subsets

We obtained RNA-seq data from FACS-sorted B cell populations from two publicly available bulk mRNA-seq datasets.^44^^,^^49^ The Agirre et al.^49^ (B-AG) B cell dataset comprised DZ, LZ, naive B (NB), MB, PB, and BMPCs from 35 samples, while the Holmes et al. (B-HM) B cell dataset comprised DZ, LZ, NB, MB, and the full GCB from 17 samples. mRNA-seq reads were aligned to the human genome (hg38) using a splice-aware aligner, STAR Methods. Quantification of gene features in the GENCODE (v38) annotation was performed by STAR Methods, while ERE expression of 14,896 HERV and 13,545 LINE elements was quantified with Telescope.^50^ As a filtering criterion, we included elements with >5 reads in at least 10% of the samples, leaving 1,464 HERVs and 1,939 LINEs in the B-HM dataset, and 1,118 HERVs and 1,520 LINEs in the B-AG dataset (Table S1).

**Figure 1:**
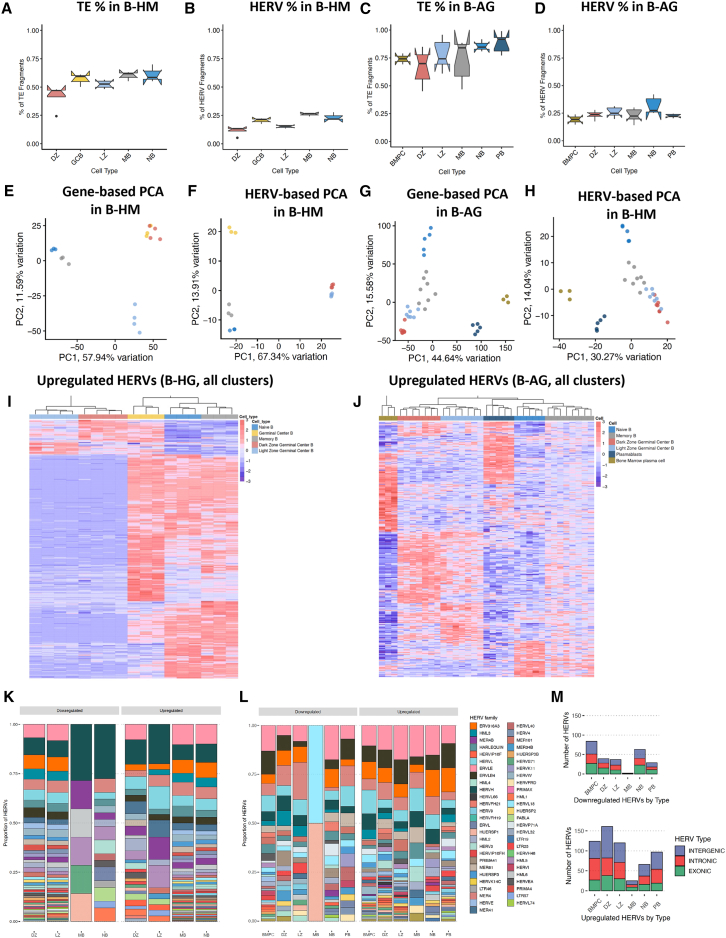
HERVs distinguish specific B cell subsets (A and B) (A) ERE reads, and (B) HERV reads as a percent of all filtered sequencing reads per cell-type in the B-HM dataset. (C–E)(C) ERE reads, and (D) HERV reads as a percent of all filtered sequencing reads per cell-type in the B-AG dataset (E) PCA plot of germinal center B cells from the Holmes dataset (NB, MB, DZ, LZ, and whole GCB), clustered by genes from the hg38 human genome annotation. (F) PCA plot of germinal center B cells from the Holmes dataset, clustered by HERV expression using the Telescope annotation. HERV expression uniquely distinguishes B cell subsets compared to genes, with HERVs in the light zone and dark zone following similar patterns of expression. (G) PCA plot of germinal center B cells from the Agirre dataset (NB, MB, DZ, LZ, PB, and BMPB), clustered by genes from the hg38 human genome annotation. (H) PCA plot of germinal center B cells from the Agirre dataset, clustered by HERV expression using the Telescope annotation. (I) Heatmap of top upregulated HERVs by cell-type in the Holmes dataset (*p*-value <0.001, log2fold change >1.5). Light zone and dark zone display downregulation of HERVs that are most highly expressed in other cell-types. (J) Heatmap of top upregulated HERVs by cell-type in the Agirre dataset (*p*-value <0.001, log2fold change >1.5). Light zone and dark zone display downregulation of HERVs that are most highly expressed in other cell-types, with plasmablasts and bone marrow plasma cells displaying the highest number of differentially expressed HERVs. (K and L) Relative abundance of HERV families upregulated and downregulated per cell-type in the Holmes and Agirre datasets, displaying a high number of loci assigned to ERVLE, HERVH, ERV316A3, HARLEQUIN, ERVLB4 and HERVFH21. (M) Number of upregulated HERVs in cell-types in the B-AG dataset, colored by the location of HERVs in relation to nearby genes (exonic, intergenic, intronic).

ERE activity in healthy B cells, including GC cells, was used for comparison with B cell lymphoma. In both B-HM and B-AG, NB cells had the highest percentage of reads assigned to EREs (0.61%, 0.74%), followed by MB cells in B-HM (0.6%), and by PB (0.88%) and BMPC (0.86%) in B-AG (Figures 1A and 1B). In both datasets, DZ had the lowest ERE expression (0.41% in B-HM and 0.71% in B-AG). Plasmablasts (PBs) and BMPCs had the lowest HERV expression despite having the highest ERE expression, indicating that a larger proportion of their ERE fragments came from non-HERV elements (Figures 1C and 1D).

We performed an unsupervised principal component analysis (PCA) to visualize sample placement based on canonical gene or HERV expression in B cell subpopulations in the B-HM (Figures 1E and 1F) and B-AG datasets (Figures 1G and 1H), using the top 90% of highly variable features within each feature type. Similar to the gene-driven PCA, the first principal component of an HERV-driven PCA in the B-HM dataset separated the NB and MB cells from LZ and DZ cells (Figure 1F). While the second principal component in the gene-driven PCA separated the LZ and DZ, the HERV expression in LZ and DZ was comparatively similar, leading to closer clustering. In the B-AG dataset, the first principal component segregated the PB and BMPC from LZ, DZ, MB, and NB, while the second principal component separated the NB, MB, LZ, and DZ (Figure 1G). Analogous to the B-HM dataset, HERV expression was more similar between LZ and DZ than gene expression (Figure 1H). The landscape of ERE activity at the RNA level in the GC B changes throughout B cell differentiation.

Next, we identified unique sets of significantly differentially expressed (DE) HERVs in each B cell subtype (Table S2). The cell subtypes with the highest number of upregulated HERVs were observed in the NB, MB, and GCB in the B-HM dataset (Figures 1I and S1A–S1F) and in the BMPC, PB, and DZ subsets in the B-AG dataset (Figures 1J and S2A–S2F). In both datasets, the most DE loci belonged to the ERVLE, HERVH, ERV316A3, ERVLB4, and MER4 families (Figures 1K and 1L). Interestingly, HERVs along the 22q11 locus such as HUERSP3B_22q11.22 and ERVLE_22q11.22b were commonly upregulated in the DZ, suggesting changes in nucleosomal accessibility at this site. HARLEQUIN_1q32.1, which has previously been found to be differentially expressed in prostate, breast, and colon cancers, was downregulated in the DZ and upregulated in the PBs and BMPCs compared to other B cell subtypes.^51^ PBs, which have been hypothesized to be the COO of ABC-DLBCL, displayed upregulation in 3 HERVP71A loci among the top DE-HERVs (Figures S3 and S4). Collectively, these data suggest significant changes in HERV loci expression can be correlated to B cell fate within the GC.

### Lymphoma subtypes have distinct HERV expression landscapes

**Figure 2:**
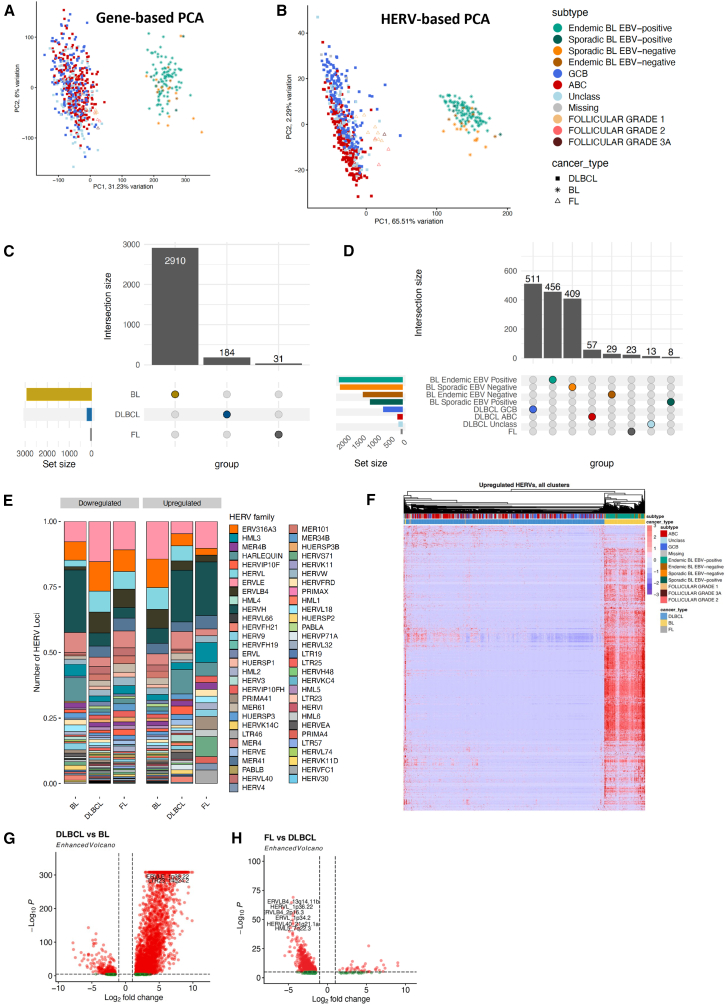
HERV expression is specific to lymphoma subtypes (A) PCA plot of 529 DLBCL samples from the TCGA and NCICCR datasets, 113 BL samples from CGCI, and 12 FL samples, clustered by genes from the hg38 human genome annotation. (B) PCA plot of 529 DLBCL samples from the TCGA and NCICCR datasets, 113 BL samples from CGCI, and 12 FL samples, clustered by HERV expression from the Telescope annotation. (C) Upset plot of the number of unique and shared HERVs upregulated in each cancer type (*p* < 0.001, log2fold change >1.5). Within the three non-Hodgkin’s B cell lymphomas, Burkitt lymphoma displays the highest HERV upregulation. (D) Upset plot of the number of unique and shared HERVs upregulated in each cancer sub-type, including ABC, GCB, and unclassified DLBCL, sporadic and endemic BL by EBV status, and follicular lymphoma. (E) Relative abundance of HERV families per lymphoma type, displaying a high number of loci assigned to ERVLE, HERVH, ERV316A3, HERVL, ERVLB4,and HERVFH21. (F) Heatmap of upregulated HERVs in each lymphoma subtype (*p* < 0.001, log2fold change >1.5), showcasing a remarkable upregulation of HERVs in BL compared to DLBCL and FL. (G) Volcano plot of differentially-expressed HERVs in DLBCL and BL (*p*-value <0.001, log2fold change >1.5. H. Volcano plot of differentially expressed HERVs in FL and DLBCL (*p*-value <0.001, log2fold change >1.5.

Since HERV expression profiles are unique to tissue sites^8^^,^^52^^,^^53^ and patterns of malignancy, we hypothesized that different B cell lymphomas would display unique HERV signatures that could be used to further classify malignancy subtypes. We obtained a total of 654 bulk RNA-seq lymphoma samples, including 529 DLBCL samples from the NCICCR and TCGA datasets, 113 EBV-positive and EBV-negative pediatric BL from CGCI, and 12 FL from CGCI. BL had the highest percentage of reads assigned to EREs and HERVs (2.27% and 0.65%), followed by FL (0.61% and 0.24%), and DLBCL (0.49% and 0.2%) (Figure S5). By conducting unsupervised clustering using the 90% most variable HERVs or canonical genes, we found that HERVs (Figure 2B) better segregate FL, ABC, EBV+ BL, EBV negative BL, GCB, and unclassified DLBCL cases than canonical genes (Figure 2A). Further characterization of lymphoma types showed that BL had 2910 uniquely upregulated HERV loci compared to DLBCL and FL, which had 184 and 31 uniquely upregulated HERVs compared to the other lymphoma subtypes respectively (Figures 2C–2F). Within the lymphoma subtypes, GCB-DLBCL had the highest number of uniquely upregulated HERVs at 511, followed by endemic EBV+ BL at 456 loci, and sporadic EBV negative BL at 409 loci (Figure S6A). When accounting for shared upregulated loci, BL exhibited broad upregulation of HERVs across all subtypes when compared to DLBCL and FL (Figures 2F–2H). Similar to benign B cells, the highest number of differentially expressed loci belonged to the ERVLE, ERV316A3, HERVH, ERVLB4, HERVL, HERVFH21, HML3, and HARLEQUIN families, with the highest upregulation of an HERV family being that of HERVH in GCB-DLBCL (Figures 2E and S6C). We also observed HERV-based DZ markers being broadly upregulated in BL compared to other lymphoma subtypes, such as MER61_3q13.11, HERV3_14q32.33, and HARLEQUIN_19p12b (Figures S4 and S7). A key HERV marker of PB and BMPCs, HARLEQUIN_1q32.1, was significantly upregulated in a subset of ABC-DLBCLs (*p* < 0.001, Figure S8). Collectively, these data demonstrate that HERVs act as cell type-specific markers that can be used to discriminate heterogeneity between B cell malignancies.

### A subset of HERV features differentiate lymphoma subtypes and GC-B COO

**Figure 3:**
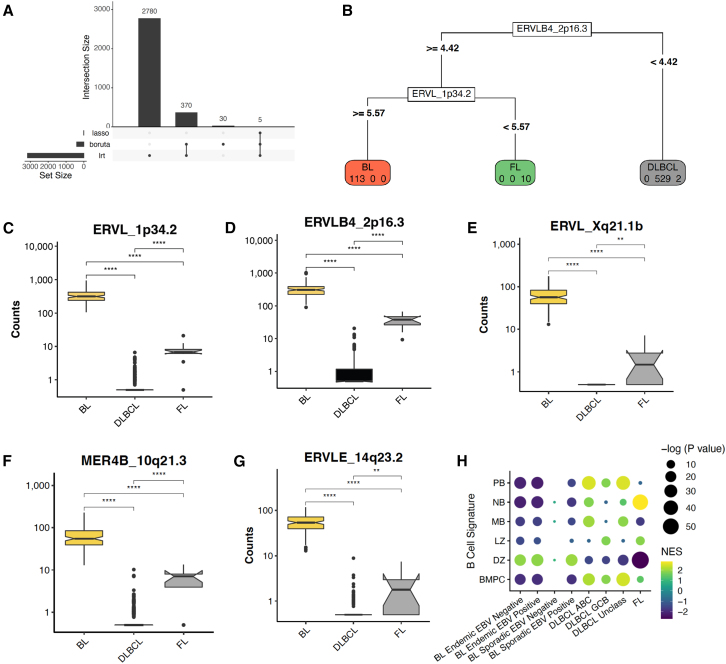
HERV expression aids in identifying lymphoma subtypes and potential GC B COO (A) UpsetR plot displaying the number of features selected by DESeq2 lowest likelihood ratio (LTR), the random forest classification with the Boruta algorithm, and the randomized least absolute shrinkage and selection operator (LASSO) regression, with 5 features being selected by all three methods. (B–G) (B) A subset of four HERVs can independently categorize lymphoma subtypes, with (C) ERVL_1p34.2 expression differentiating between BL and FL, and (D) ERLB4_2p16.3 differentiating between DLBCL, and FL and BL, in addition to (E) ERVL_Xq21.1b, (F) MER4B_10q21.3, and (G) ERVLE_14q23.2. (H) Correlation plot of lymphoma sub-types with gene and HERV- based B-cell-of-origin signatures. Signature gene sets were created using a subset of the top 150 and top 25 upregulated genes and HERVs per cell-type from the Agirre 2019 B cell dataset.

We next asked whether the HERV-driven B cell malignancy signatures could complement gene expression data to best define the GC COO. Our goal was to reduce the large number of DE HERV features to the lowest possible targets for reliable classification. Including only DE HERVS with an FDR <0.001 and log2fold >1.5, we used two unsupervised feature selection methods: (1) the random forest classification with the Boruta algorithm^54^ and (2) the randomized least absolute shrinkage and selection operator (LASSO) regression,^55^ identifying just 5 HERVs to differentiate between DLBCL, BL, and FL (Figure 3A). Out of the 5 HERVs, ERVL_1p34.2 expression differentiated between BL and FL, while ERLB4_2p16.3 differentiated between DLBCL, and FL and BL (Figures 3B–3G). We next created feature sets for each B cell subtype from the B-AG dataset, using the top 150 upregulated genes and top 25 upregulated HERVs for MB, NB, DZ, LZ, PB, and BMPC (Table S3). To assign COO, we ran a fast HERV and gene set enrichment analysis (F-HAGSEA) using an adaptive multilevel split Monte Carlo method.^56^ Consistent with known literature,^26^ we found that all BL subsets were enriched in DZ signatures, ABC-DLBCL enriched in PB and MB signatures, GCB-DLBCL in LZ, and, interestingly, FL in NB and LZ (Figure 3H). GCB-DLBCL were also enriched for PB signatures, suggesting a higher heterogeneity within the GCB-DLBCL samples originating from intermediate and late-stage selection within the LZ. Overall, our findings indicate that HERVs are uniquely expressed in healthy B cells and lymphoma subtypes, and that HERV expression profiles can be further used in combination with gene expression profiles to distinguish the COO for B cell malignancies.

### Seven distinct HERV signatures categorize diffuse large B cell lymphoma

**Figure 4:**
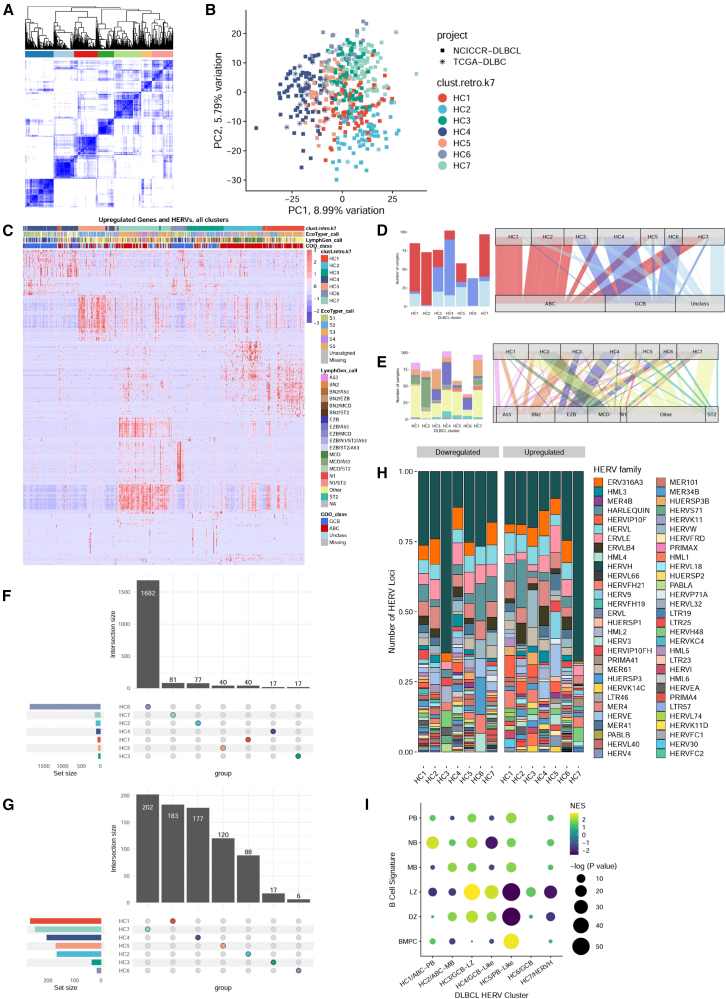
Seven distinct HERV signatures in diffuse large B-cell lymphoma (A) Consensus clustering of TCGA and NCICCR DLBCL samples find seven distinct sample clusters, based on expression values of the top 10% of most variable HERVs. (B) PCA of DLBCL samples, colored by HERV clusters. (C) Alluvial diagram showcasing HERV cluster assignment in comparison with recent DLBCL classification paradigms, including the LymphGen, EcoTyper, DBL Hit presence, and classic cell-of-origin classifications. When comparing HERV clusters to the COO subtypes, HC1 and HC2 belong predominantly to the ABC-DLBCL class, while HC4 and HC6 belong predominantly to the GCB-DLBCL class. HC3 and HC5 are mixed clusters of all three classifications, while HC7 encompasses ABC-DLBCL, with the highest number of unclassified samples. (D) When comparing HERV clusters to the LymphGen classes, HC2 consists predominantly of MCD, HC3 consists of the highest number of BN2, and HC4 and HC6 encompass the highest number of EZB. The N1 subclass is split between HC5 and HC7. (E) Heatmap of the top 50 upregulated genes and HERVs per DLBCL cluster (*p* < 0.001, log2fold change >1.5). (F) Upset plot of the uniquely upregulated HERVS per cluster, and (G) Upset plot of the uniquely downregulated HERVs per cluster, finding the highest number of unique genes in C6. (H) Relative abundance of HERV families per DLBCL type. (I) Gene and HERV-driven B-cell-of-origin classification of each HERV-driven DLBCL cluster. Signature gene sets were created using a subset of the top 150 and top 25 upregulated genes and HERVs per cell-type from the Agirre 2019 B cell dataset. HC1 and HC2, which belong predominantly to the ABC-DLBCL subclass, are enriched in NB and PB, and MB and DZ gene-sets respectively. HC3, which is a mixed subtype, is most enriched in LZ signatures. HC4 and HC6, which are both predominantly GCB-DLBCLs, are enriched in LZ signatures. HC5 and HC7, which are mixed subtypes containing ABC-DLBCL and unclassified samples, are most enriched for BMPC signatures, with negative enrichment scores for both LZ and DZ.

Given that ABC-DLBCL and GCB-DLBCL display distinct patterns of HERV expression, we investigated whether subsets within the COO classes possessed unique HERV signatures that could further define their characterization. We performed unsupervised consensus clustering with ConsensusClusterPlus^57^ based on DE HERVs to identify the number of potential subsets, *k*, along with the strength of each sample’s membership in the identified class. While the most stable *k* yielded 3 clusters most consistent with the pre-existing COO classes of ABC-DLBCL, GCB-DLBCL, and unclassified DLBCL, we chose a *k* of 7 to potentially identify sub-classes of HERV signatures within the ABC, GCB, and unclassified DLBCLs (Figures 4A–4C). When comparing HERV clusters (HCs) to the COO subtypes, the ABC-DLBCL were split predominantly into HC1 and HC2, while HC4 and HC6 belonged predominantly to the GCB-DLBCL class. HC3 and HC5 were mixed clusters of all three classifications, while HC7 encompassed ABC-DLBCL and the highest number of unclassified samples (Figures 4D and S9A). When compared to the LymphGen classes, HC2 consisted predominantly of MCD, HC3 contained the highest number of BN2, and HC4 and HC6 encompassed the highest number of EZB. The N1 subclass was split between HC5 and HC7 (Figures 4E and S9A). HC6 had the highest number of uniquely upregulated HERVs at 1,682 loci, while HC7 had the highest number of uniquely downregulated HERVs, at 202 loci (Figures 4F and 4G). Compared to healthy B cells, loci from the HERVH family represented a higher proportion of upregulated HERVs (Figure 4H), with HC7 displaying the highest upregulation of HERVH transcripts. Four key HERVs that could differentiate the DLBCL clusters (Figure S10A) were HERVH_16p13.2e, HERVW_2q23.3, HML2_7p22.1, and HERVH_7q11.23a (Figures S10B–S10E). HERVH_16p13.2e differentiates HC7 from the remaining clusters, while HERVH_16p13.2e differentiates HC1 and HC2. HML2_7p22.1 separates HC4 and HC6 from HC3, HC4, and HC7, and then further differentiates within the clusters.

To determine potential GC-B COO for the seven DLBCL subsets, we conducted an F-HAGSEA analysis against the B cell signatures, using feature ranks derived from DESEq2 differential testing^58^ (Figure 4I). HC1 and HC2 were most enriched in NB and PB, and MB and DZ gene-sets respectively. HC3, which is a mixed subtype, was most enriched in LZ signatures. HC4 and HC6, which are both predominantly GCB-DLBCLs, were also enriched in LZ signatures. HC5 and HC7 were most enriched for BMPC signatures, with negative enrichment scores for both LZ and DZ. We thus designated HC1 and HC2 with the names “ABC-PB” and “ABC-MB” (Figure S11), HC3, HC4 and HC6 with the names “GCB-LZ”, “GCB-Like”, and “GCB” (Figure S12), HC5 with the name “PB-Like”, and HC7 with “HERVH” (Figure S13). Overall, our results identified 7 distinct HERV signatures in DLBCL samples which identify additional subclasses of the currently implemented DLBCL COO classifications.

### Two distinct HERV signatures are found in Burkitt lymphoma that are indicative of EBV status

**Figure 5:**
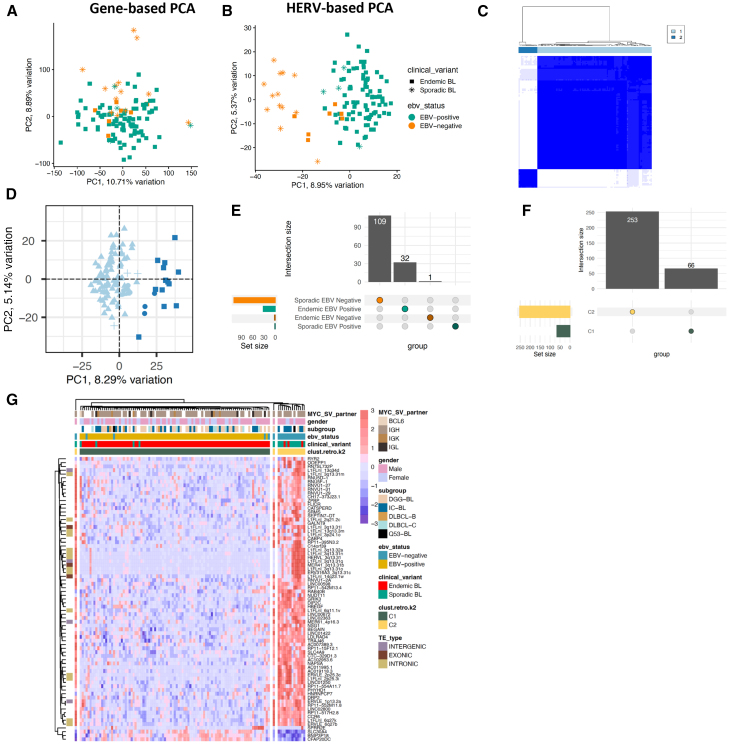
Two distinct HERV signatures are found in Burkitt lymphoma independent of EBV status (A) PCA plot of 113 BL samples from CGCI datasets, 113 BL samples from CGCI, clustered by genes from the hg38 human genome annotation. (B) PCA plot of BL samples, clustered by HERV expression from the Telescope annotation. HERV-only clustering reliably separates the EBV-positive and EBV-negative samples, showcasing distinct expression patterns in the HERVs that are not captured with gene-only clustering. (C–E) (C and D) Consensus clustering of BL samples find two distinct sample clusters, with BL-C1 containing all EBV-positive endemic and sporadic BL samples, along with three EBV-negative endemic BL samples. BL-C2 consists of all EBV negative sporadic BL samples, along with three EBV-negative endemic BL samples (E) BL-C2, which predominantly contains EBV negative sporadic BL samples, contains 253 uniquely upregulated HERVs, compared to 66 in BL-C1. (F) When comparing within subtypes, EBV- sporadic BL has the most number of uniquely upregulated HERVs, followed by EBV+ endemic BL. (G) Heatmap of the top 50 upregulated genes and HERVs per DLBCL cluster (*p* < 0.001, log2fold change >1.5).

**Figure 6:**
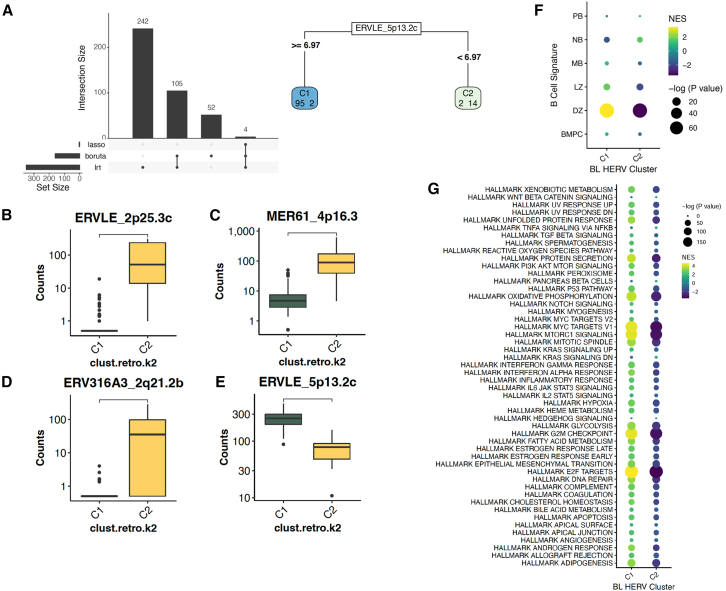
BL subtypes and EBV status have distinct biological and HERV signatures (A–E) (A) Feature selection of differentially-expressed HERVs per cluster using DESeq2 LRT, Boruta, and Lasso find 4 HERVs sufficient to distinguish between BL-C1 and BL-C2, including (B) ERVLE_2p25.3c, (C) MER61_4p16.3, (D) ERV316A3_2q21.2b, and (E) ERVLE_5p13.2c. (F) Gene and HERV-driven B-cell-of-origin classification of each HERV-driven BL cluster. Signature gene sets were created using a subset of the top 150 and top 25 upregulated genes and HERVs per cell-type from the Agirre 2019 B cell dataset. BL-C1 displays an enrichment of DZ gene-sets compared to BL-C2. (G) Enrichment of hallmark pathways for the two HERV clusters, showcasing an overall upregulation in BL-C1 compared to BL-C2 for MYC targets, E2F targets, and epithelial mesenchymal transition.

Since HERVs are transactivated by EBV,^28^ we hypothesized that heterogeneous HERV expression profiles in BL are driven by infection with EBV. We performed unsupervised PCA clustering of pediatric BL samples based on gene expression (Figure 5A) and HERV expression (Figure 5B) alone. Surprisingly, we found that while the gene-based PCA did not segregate samples by EBV status, HERV expression separated BL status into EBV+ and EBV- clusters. To confirm the results of the PCA, we performed consensus clustering of samples based on HERV expression, finding the most stable clusters with a *k* of 2 (Figures 5C and 5D). The BL cluster 1 (BL-C1) was composed entirely of EBV- samples (13 EBV- endemic BL samples and 3 EBV- sporadic BL samples) while BL cluster 2 (BL-C2) was composed of primarily EBV positive samples (4 EBV- endemic BL samples, 4 EBV+ sporadic BL, and 89 EBV+ endemic BL). Collectively, these separations were driven by an overall upregulation of TEs in BL-C2 (Figures 5E–5G), with 253 uniquely upregulated HERVs in BL-C2, compared to 66 in BL-C1 (Figure S14A). We next sought to identify the HERV signatures driving separation of BL-C1 and BL-C2 with the Boruta algorithm, LASSO regression, and the likelihood ratio test (LRT) provided by DESEQ2. In doing such, we identified a subset of four HERVs that further distinguished between the BL-C1 and BL-C2 (Figure 6A). Amongst all HERVs, we identified ERVLE_2p25.3c (Figure 6B), MER61_4p16.3 (Figure 6C), ERV316A3_2q21.2b (Figure 6D), and ERVLE_5p13.2c (Figure 6E) as definitive markers that distinguished between the entirely EBV- BL-C1, and the largely EBV+ BL-C2 (Figure S15). We further identified BL-C1 to have a more distinct DZ signature compared to BL-C2, and additionally found a higher relative upregulation of Hallmark pathways identified by the Molecular Signatures Database (MsigDB)^59^^,^^60^ when compared to BL-C2 (Figures 6F and 6G). Collectively, these results demonstrate that EBV status is a major determinant of HERV expression in BL subtypes, and that the expression of HERVs can be applied to better define the heterogeneity of pediatric BL.

### HERV expression is linked with survival outcomes in DLBCL

**Figure 7:**
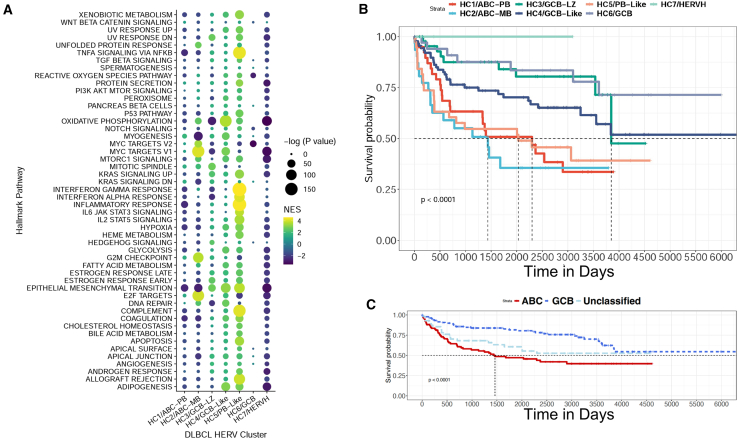
HERV-driven DLBCL subtypes have distinct biological properties and survival outcomes (A) Enrichment of hallmark pathways for the seven HERV clusters, showcasing distinct enrichment patterns for each cluster. HC1, which contains predominantly ABC-DLBCL and is enriched for NB, PB, and BMPC signatures, displays an overall downregulation of most hallmark pathways. HC2, which contains predominantly ABC-DLBCL and is enriched for MB and DZ signatures, shows the highest enrichment for MYC targets V1, G2M checkpoint, and E2F targets. HC3, which is a mixed cluster with LZ signatures, shows enrichment for epithelial mesenchymal transition, mitotic spindle, and a negative enrichment for DNA repair, interferon alpha and gamma response, MYC targets, and oxidative phosphorylation. HC4, which consists predominantly of GCB-DLBCL and displays LZ and DZ signatures, is enriched in oxidative phosphorylation, MYC targets V1, epithelial mesenchymal transition, and adipogenesis. HC5, which is another mixed cluster with BMPC signatures, shows a highly significant enrichment of interferon gamma and alpha response, inflammatory response, IL6 JAK STAT3 signaling, TNFA signaling via NFKB, and IL2 STAT5 signaling. HC6 shows a negative enrichment of MYC targets V2. HC7 displays an overall negative enrichment for most pathways compared to the other clusters. (B) Survival plot of the seven DLBCL clusters, showcasing the worst prognosis for HC1/ABC-PB (*n* = 39) and HC2/ABC-MB (*n* = 31), followed by HC5/PB-Like (*n* = 38), HC4/GCB-Like (*n* = 92), HC3/GCB-LZ (*n* = 45) and HC6/GCB (*n* = 34), and HC7/HERVH (*n* = 3). (C) Survival plot of the original DLBCL cell-of-origin classifications, showing the worst prognosis for ABC-DLBCL, followed by Unclassified-DLBCL, and GCB-DLBCL.

Finally, we hypothesized that the seven HERV-driven DLBCL subclasses with distinct predictive COO would display transcriptomic differences that correlate with their prognostic outcome. We implemented an FGSEA analysis with the Hallmark pathways collected from MsigDB to calculate broad phenotypic alterations between our COO subtypes (Figure 7A). HC1/ABC-PB displayed an overall downregulation of most Hallmark pathways, although HC2/ABC-MB, which was enriched for MB and DZ signatures, showed the highest enrichment for the “MYC targets V1”, “G2M checkpoint”, and “E2F targets” pathways. HC3/GCB-LZ displayed enrichment for “epithelial mesenchymal transition”, “mitotic spindle”, and a negative enrichment for the “DNA repair”, “interferon alpha and gamma response”, “MYC targets V1”, “MYC targets V2”, and “oxidative phosphorylation” pathways. HC4/GCB-like was enriched in “oxidative phosphorylation”, “MYC targets V1”, “epithelial mesenchymal transition”, and “adipogenesis” pathways, while HC6/GCB displayed a negative enrichment of “MYC targets V1” and “MYC targets V2” pathways. HC7/HERVH displayed an overall negative enrichment for most Hallmark pathways compared to the other clusters. The HC5/PB-Like showed a highly significant enrichment of the “interferon gamma and alpha response”, “inflammatory response”, “IL6 JAK STAT3 signaling”, “TNFA signaling via NFKB”, and “IL2 STAT5 signaling” pathways. Overall, samples from the HC5/PB-Like cluster had the highest enrichment for pathways indicating changes in local immunity (Figure S16), including “Cytotoxic T-lymphocyte-associated protein 4 (CTLA4)”, “TCR”, “IL17”, “IL10”, and “IL12”.

We performed a Kaplan-Meier analysis to examine the relationship between HERV clusters and clinical outcomes, in comparison with previous COO classifications (Figures 7B and 7C). Consistent with previous findings,^61^ ABC-associated groups had the shortest long-term survival. Groups with the worst prognoses were HC1/ABC-PB (*n* = 39) and HC2/ABC-MB (*n* = 30), followed by HC5/PB-Like (*n* = 37), HC4/GCB-Like (*n* = 89), HC3/GCB-LZ (*n* = 45), HC6/GCB (*n* = 34), and HC7/HERVH (*n* = 3) (Figure 7B). Importantly, when implemented on the same cases denoted as ABC-DLBCL, GCB-DLBCL, or unclassified, the HERV-based classifications identified patient subsets that significantly correlated with prognostic outcomes. Patients in the HC5/PB-Like cluster (43% ABC, 45% Unclassified, 12% GCB) had a survival outcome much closer to the ABC-like clusters HC1 and HC2, despite having a large proportion of unclassified and GCB diagnoses. Similarly, prognostic values of previously unclassified DLBCLs had a significant range of favorable to unfavorable outcomes (Figure S17). To assess the involvement of known DLBCL mutations and translocations in our clusters, we stratified the DLBCL samples by cluster and calculated the enrichment of key mutations, including *TP53*, *CD79B*, *MYD88*, *NOTCH2*, *NOTCH1*, *EZH2*, *CD58*, *TET2*, and *SK1*, as well as major translocations involving *BCL6*, *BCL2*, and *MYC* (Figure S18A). Our analysis revealed that HC1/ABC-PB and HC2/ABC-MB were highly enriched for *CD79B* mutations, with HC2/ABC-MB also showing significant enrichment for *MYD88* mutations (Figure S18B). This aligns with our observation that HC1/ABC-PB and HC2/ABC-MB contained the majority of ABC-DLBCL samples, with HC2 being particularly enriched for MCD-DLBCL cases. Additionally, HC3/GCB-LZ was significantly enriched for *BCL6* translocations, while HC4/GCB-Like exhibited the highest enrichment for *EZH2* mutations along with *BCL2* and *MYC* fusions. Finally, HC5/PB-Like and HC7/HERVH were notably enriched for mutations affecting the *NOTCH1* signaling pathway.

Taken together, our HERV-based clusters both recapitulate known transcriptional and mutational DLBCL subclasses, while offering insights on distinct biological subtypes of DLBCL that may be influenced by both genetic and non-genetic factors, such as the tumor microenvironment, immune interactions, or chromatin accessibility. Overall, DLBCL subclasses based on HERV signatures were able to be predictive of prognostic outcomes within the ABC-DLBCL, GCB-DLBCL, and unclassified-DLBCL cases.

## Discussion

In this study, we developed a comprehensive locus-specific map of ERE expression in human GC B cells and in B cell malignancies which arise from the GC. By characterizing ERE activity in the healthy GC and associated B cell lymphomas, we identified HERV expression profiles specific to GC reaction stages and used them to further classify the COO in B cell malignancies. ERE expression levels range throughout the GC reaction, with high expression in NB, MB, PB, and BMPC cells, moderately high expression in the LZ, and low expression in the DZ. Despite having relatively high ERE transcription, PB and BMPC had the lowest percent of HERV fragments. Interestingly however, the PB and BMPC possessed the highest number of uniquely upregulated HERV loci. Despite HERV expression representing under 1% of the coding and non-coding transcriptome, our analysis generated a signature based on 11 HERV markers to accurately classify GC B cell subtypes.

We next found that leveraging HERV transcripts with gene expression improved upon the lymphoma type classification when compared to gene expression alone, particularly between DLBCL and FL. In accordance with previous findings, BL samples most closely resembled the DZ, GCB-DLBCL resembled the LZ, ABC-DLBCL corresponded with MBs, PBs, and BMPCs, and FL corresponded with the LZ and NBs. We also found specific HERV markers of GC B cell types upregulated in B cell lymphomas arising from their COO, including the DZ-associated element HARLEQUIN_19p12.b as a key marker of BL. Similarly, HARLEQUIN_1q32.1, which is a PB-associated HERV, is a key marker of ABC-DLBCL and has been previously implicated in prostate, breast, and colon cancers.^51^

We also used HERV signatures to expand the current COO classifications from three to seven subsets, with each corresponding to single or mixed B cell subtypes from the GC. Together, the two ABC-like clusters represented precursors to PBs and MBs. Recent findings have reported intermediate phases of the GC between the LZ and DZ compartmentalization,^44^ in addition to MB precursors which are reflected in a fraction of DLBCLs.^62^ These studies are consistent with our findings of “mixed” DLBCLs with competing gene and HERV signatures, particularly HC2/ABC-MB, which had an MB-like signature and encompassed many MCD-DLBCL cases.

Interestingly, a key feature of our classification system for DLBCLs was the expression of HML2_7p22.1, a largely intact HERV provirus which contains an open reading frame (ORF) for a fusogenic retroviral envelope gene.^52^^,^^63^^,^^64^ HML2_7p22.1 is one of two elements from the HML2 family that possess an intact envelope.^52^ While HML2_7p22.1 is expressed in 15 different human tissue types,^52^ it has also been implicated for its fusogenic activity in melanoma cell lines^65^ and may be immunosuppressive in nature.^66^

Our HERV-based clustering identified additional sub-clusters within the GCB-like and unclassified DLBCL cases that demonstrate distinct survival outcomes. Briefly, the ABC-like clusters predictably had the least favorable prognostic outcomes. The GCB-like clusters displayed greater range than what would have been defined as a single class, with the HC3/GCB-Like cases having worse survival outcomes compared to HC4/GCB-LZ and HC6/GCB. Cases from the HC5/PB-Like cluster, which is likely to originate from intermediate phases of the GC reaction, had unfavorable survival outcomes, second only to the ABC-like clusters. Differences in clinical outcome may be attributed to the drastic changes observed in immune signatures within this cluster. The HC7/HERVH cluster lacked survival data to draw definitive conclusions and therefore requires further investigation. However, this cluster demonstrated a clear downregulation of most hallmark pathways expressed in the majority of our DLBCL clusters and is likely phenotypically distinct.

Collectively, our analysis of healthy GC-B cells and B cell lymphomas suggests that malignant cells retain transcriptomic signatures from their COO. The observed increase in HERV transcripts in cancerous tissue, particularly BL, suggests a change in the epigenetic state of the B cell derived COO in relation with infection status. This is relevant for other EBV and HIV-1 associated B cell lymphomas as well, where infection status may promote differential patterns of HERV expression. Overall, the predictive capabilities of the HERV-driven lymphoma clustering suggest a further need to understand the regulatory, transcriptional, and post-transcriptional activity of these EREs in healthy and malignant tissues. HERV expression in the healthy GC and lymphomas could function as a diagnostic tool for B cell malignancies whose unique properties provide targets exploitable for developing therapeutic interventions.

### Limitations of the study

While our findings provide valuable insights into HERV expression in DLBCL, several limitations remain. Our study is limited by cohort size, which restricts the strength of survival analyses and larger patient cohorts are needed to better assess the significance of their expression on prognostic outcomes. Our analyses also rely purely on RNA sequencing, and it is therefore unknown whether any HERV elements of interest possess biological activity at the protein level. Our study more specifically relied solely on bulk RNA sequencing approaches, and single-cell RNA sequencing may further refine our understanding of HERV activity in the GC and B cell lymphomas by identifying rare and lowly abundant cell states. Further investigation is also needed to determine the biological roles of differential HERV expression, such as whether increased HERV expression correlates with specific immune phenotypes, which could reveal potential interactions between HERV activity and the tumor microenvironment. Other limitations of this study regard any potential mechanisms responsible for differential HERV expression. While we show locus level HERV expression specific to healthy and malignant B cell subtypes, the molecular or cellular mechanisms responsible for these changes are indeterminable with this approach. Lastly, a major limitation of this study regards the quantification of repetitive elements such as EREs. Repetitive element quantification is a computationally challenging task magnified in short-read mRNA sequencing data.^67^^,^^68^^,^^69^^,^^70^ While several tools have been developed to quantify these elements, we chose to use Telescope, which uses an expectation maximization algorithm to reassign multi-mapping ambiguous reads by borrowing strength from neighboring alignments. Cross-validating these findings with multiple datasets, as well as benchmarking these results different quantification tools and annotations, such as those provided by SQuIRE^71^ and ERVmap,^72^ can help to confirm or deny the putative roles of ERE activity in hematological malignancies.

## Resource availability

### Lead contact

All further information, requests for access to resources, or clarifications should be directed to and will be fulfilled by the lead contact, Dr. Matthew Bendall (mlb4001@med.cornell.edu).

### Materials availability

This study did not generate new or unique reagents.

### Data and code availability

• This study utilized only publicly available data.
• All original code used in this study has been deposited on GitHub and published with Zenodo. Custom and reproducible Snakemake (v7.14.0) pipelines were created for the DLBCL (https://doi.org/10.5281/zenodo.15256968), BL (https://doi.org/10.5281/zenodo.15256978), FL (https://doi.org/10.5281/zenodo.15256975), and healthy B cell datasets (https://doi.org/10.5281/zenodo.15256973), separated by the source of data access. Input samples were supplied through the config.yaml file for each pipeline, which also contained consistent parameters for data processing. The same package versions were used for gene and ERE quantification in each Snakemake pipeline.^73^ All downstream analysis was conducted in R (v4.0.2), and can be accessed on GitHub (https://doi.org/10.5281/zenodo.15256016). Any additional information to assist in reanalyzing the data provided in this paper are available upon request from the lead contact.
• These accession numbers for the public datasets are listed below, in addition to the key resources table.^44^^,^^45^^,^^49^^,^^74^^,^^75^ Samples belonging to the B-AG (n = 35; GEO: GSE114816) and B-HM (n = 17; GEO: GSE139833) datasets were downloaded as FASTQ files using fasterq-dump from the SRA toolkit. RNA-seq data from the HIV- DLBCL samples (n = 529) belonging to the TCGA and NCICCR research programs were obtained via the Genome Data Commons (dbGaP). Samples datasets were downloaded as FASTQ files using fasterq-dump from the SRA toolkit. RNA-seq data from the HIV- DLBCL samples (n = 529) belonging to the TCGA and NCICCR research programs were obtained via the dbGaP accession “phs001444.v2.p1”. The BL samples (n = 113) were obtained as part of CGCI’s Burkitt Lymphoma Genome Sequencing Project (BLGSP), and accessed via dbGaP: phs000235.v16.p4. The FL samples (n = 12) were obtained as part of CGCI’s Non-Hodgkin Lymphoma - Follicular Lymphoma (NHL - FL) initiative and accessed through SRA toolkit via dbGaP: phs000235.v7.p2. Clinical, demographic, and survival metadata was obtained via the TCGABiolinks R package (v2.18.0). LymphGen,^47^ EcoTyper,^48^ Chapuy et al.,^74^ and Holmes et al.^44^ DLBCL classification calls were obtained from the respective publications.

## Acknowledgments

The work was supported in part by US National Institutes of Health (NIH) grant NCI CA260691 (D.F.N.) and NIAID UM1AI164559 (D.F.N.). MLB is supported in part by the 10.13039/100019688Department of Medicine Fund for the Future program at Weill Cornell Medicine sponsored by the 10.13039/100019664Elsa Miller Foundation. J.L.M. was supported in part by a Medical Scientist Training Program grant to the Weill Cornell–Rockefeller–Sloan Kettering Tri-Institutional MD-PhD Program (T32GM007739), and by a grant from the 10.13039/100002224Melanoma Research Foundation CK0041482163, generously supported by the Silverstein family.

The results shown here are in whole or part based upon data generated by the TCGA Research Network: https://www.cancer.gov/tcga, including data generated by the Cancer Genome Characterization Initiative (phs000235), developed by the National Cancer Institute. Information about CGCI projects can be found at https://ocg.cancer.gov/programs/cgci. The Genomic Variation in Diffuse Large B-cell Lymphomas study was supported by the 10.13039/100030692Intramural Research Program of the 10.13039/100000054National Cancer Institute, National Institutes of Health, Department of Health and Human Services. The datasets have been accessed through the 10.13039/100000002NIH database for Genotypes and Phenotypes (dbGaP).

We would like to acknowledge helpful discussions with members of the Cesarman, Feschotte, and Leal labs, in addition to the overall HERV Lymphoma team.

The graphical abstract was created in BioRender. D.F.N. (2025) https://BioRender.com/q33lbri.

## Author contributions

Study design and conception: B.S., M.L.B., and D.F.N. Performed analyses: B.S. Wrote the paper: B.S. Provided support with data analysis and interpretation: T.F., N.D., and J.L.M. Contributed knowledge: all authors. All authors reviewed the final draft.

## Declaration of interests

P.M.: ADCT: Consultancy. All other authors declare no competing interests.

## STAR★Methods

### Key resources table

**Table:** 

REAGENT or RESOURCE	SOURCE	IDENTIFIER
**Deposited data**		

B-AG Dataset	Holmes et al.^44^	GEO: GSE114816
B-HM Dataset	Agirre et al.^49^	GEO: GSE139833
DLBCL - NCICCR	Schmidt et al.^45^	dbGaP: phs001444.v2.p1
DLBCL - TCGA	Weinstein et al.^75^	dbGaP: phs001444.v2.p1
BL – CGCI Burkitt Lymphoma Genome Sequencing Project	Weinstein et al.^75^	dbGaP: phs000235.v16.p4
FL - CGCI Non-Hodgkin Lymphoma - Follicular Lymphoma	Weinstein et al.^75^	dbGaP: phs000235.v7.p2

**Software and algorithms**		

Snakemake (v7.14.0)	Köster et al.^73^	https://snakemake.github.io/
Telescope (v1.0.3)	Bendall et al.^50^	https://github.com/mlbendall/telescope
DESeq2 (v1.30.1)	Love et al.^58^	https://bioconductor.org/packages/release/bioc/html/DESeq2.html
ConsensusClusterPlus (v1.54.0)	Wilkerson et al.^57^	https://bioconductor.org/packages/release/bioc/html/ConsensusClusterPlus.html
c060 (v0.2-9)	Sill et al.^55^	https://cran.r-project.org/web/packages/c060/index.html
Boruta (v8.0.0)	Kursa et al.^54^	https://gitlab.com/mbq/Boruta/
fgsea (v1.16.0)	Korotkevich et al.^56^	https://github.com/ctlab/fgsea
MSigDB	Liberzon et al.^60^	https://www.gsea-msigdb.org/gsea/msigdb/

**Other**		

Snakemake pipelines for TE and gene quantification	This paper	https://doi.org/10.5281/zenodo.15256968 https://doi.org/10.5281/zenodo.15256978 https://doi.org/10.5281/zenodo.15256975 https://doi.org/10.5281/zenodo.15256973
Data analyses and resources related to TE quantification, predictions, and lymphoma classification.	This paper	https://doi.org/10.5281/zenodo.15256017

### Method details

#### Transcriptomic profiling and locus-specific HERV prediction

For DLBCL and BL, downloaded BAM files were converted to FASTQ using picard-slim (v2.25). FASTQ files for all samples were then aligned to Hg38 using STAR (v2.7.9a), with parameters “--outSAMattributes NH HI NM MD AS XS --outSAMtype BAM Unsorted --quantMode GeneCounts --outSAMstrandField intronMotif --outFilterMultimapNmax 200 --winAnchorMultimapNmax 200 --outSAMunmapped Within KeepPairs”. We used Telescope (v1.0.3) for profiling ERE expression, which allows for the locus-specific identification of TEs using expectation maximization algorithm. The Telescope assign module was used with the parameters “--theta_prior 200000 --max_iter 200”, along with a custom transposable element annotation (retro.hg38.v1), accessible at https://github.com/mlbendall/telescope_annotation_db. Meta annotations for TEs with the nearest genes, gene overlaps, and the TE status of intronic, exonic, or intergenic, were obtained from https://github.com/liniguez/Telescope_MetaAnnotations.

#### Unsupervised clustering

Gene and ERE counts were first filtered, such that only features with more than 5 observations within a minimum sample threshold (5 samples for the 529 DLBCLs, 5 samples for 113 BLs, 2 samples for 12 FLs, 2 samples for 17 B-HM, and 4 samples for 35 B-AG) were retained. Normalized counts were calculated using the estimated size factors within DESeq2 (v1.30.1) and subsequently transformed using variance-stabilizing transformation. PCA was carried out on the transformed counts and then visualized using PCATools (v2.2.0). Clustering on DLBCL and BL samples was performed using ConsensusClusterPlus (v1.54.0), with 1000 repetitions. Clusters were calculated for *k* = 2 through *k* = 9, and assessed through the calculated consensus matrices, silhouette statistics, molecular and clinical indicators, and agreement with previously described classifications. Final clusters of *k* = 7 for DLBCL and *k* = 2 for BL were chosen based on the aforementioned statistical and clinical indicators. Fisher’s exact test was used to test each cluster against categorical variables and previous classifications. Alluvial plots comparing HERV clusters to previous DLBCL and Bl classifications were created using ggalluvial (v0.12.3).

#### Differential expression analysis

DE testing was performed between and within lymphoma subtypes, and separately within B-cell subtypes for the B-AG and B-HM datasets. A negative binomial model was used for DE testing, with a significance cutoff of *p* = 0.001, and a log2fold change cutoff of >1.5. B-cell subtypes were compared individually within the B-AG and B-Hm datasets, with a design of ∼cell_type +0. To compare between lymphoma types, two DE models were created, with the broad lymphoma type (∼ cancer_type + 0, where cancer_type refers to DLBCL, BL, or FL), and a narrower lymphoma subtype (∼ subtype + 0, where the subtypes included ABC-DLBCL, GCB-DLBCL, Unclassified, EBV+/- Sporadic and Endemic BL, and FL). Differential expression testing was also performed within DLBCL (∼COO + 0), BL (∼ebv_status + 0), and the unsupervised HERV clusters for DLBCL (∼ clust.retro.k7 + 0) and BL ( clust.retro.k2 + 0) respectively. Results were extracted as DESeqResults objects, with a numbered contrast of each group compared against all others. HERVs that were uniquely upregulated and downregulated per group were visualized with UpsetR (1.4.0) and ComplexUpset (1.3.3). The top n differentially expressed genes and HERVs were visualized with pheatmap (1.0.12). The significance and effect size of DE genes and HERVs were calculated and visualized with EnhancedVolcano (1.8.0).

#### HERV-based feature selection and model

Supervised learning and HERV-based feature selection was implemented as previously described.^76^ Briefly, pre-filtered HERV matrices were used for DESeq2’s likelihood ratio test (LRT), which was used to create a model of the HERV clusters for DLBCL (∼clust.retro.k7 + 1). BL (∼clust.retro.k2 + 1), and healthy B-cells from the B-AG dataset (∼cell_type +1), with a significance cutoff of FDR <0.001. Variance transformed counts from DESeq2 were extracted for feature selection with the Boruta random forest algorithm and the randomized LASSO regression. LASSO regression with stability selection was used to find the minimum optimal numbers of features defining each group, using the glmnet (v4.1-6) and c060 (v0.2-9) packages. LASSO was implemented with multinomial logistic regression with a grouped penalty, ensuring that each selected feature had multinomial coefficients of either all non-0 or all 0. Stability selection was performed with 200 subsamples, and a proportion threshold of 0.6. For a less stringent feature selection of all relevant features, we used the Boruta (v8.0.0) algorithm and the randomForest package (v4.6-12) for random classification, with ntree = 1000 and maxRuns = 1000. Final features were selected using an intersection of the three methods and visualized with UpsetR. The LASSO signature was used to create a final classification tree, with recursive partitioning implemented in rpart (v4.1.19) and rpart.plot (v3.1.1).

#### HERV- and gene-set enrichment analyses

Preranked gene-set enrichment analysis (GSEA) was performed using the fgsea package (v1.16.0), which uses an adaptive multilevel split Monte Carlo method. Fold change statistics and *p*-values from DESEq2 differential testing were used to estimate gene and HERV ranks. Overall biological signatures in BL and DLBCL HERV clusters were calculated using the Hallmark and Kyoto Encyclopedia of Genes and Genomes^77^ gene sets from MSigDB.^60^ We created custom B-cell signature gene sets, using the top 150 genes and top 25 HERVs upregulated in each B-cell subtype in the B-AG dataset, and performed a combined HAGSEA to determine potential COO of our DLBCL and BL HERV clusters. Effect size and *p*-values were visualized for the GSEA and HAGSEA using corrplot (v0.92) in R.

#### Survival analysis

Survival analysis was conducted using the survival R package (v3.1-12), using the log-rank test for group-level comparisons (rho = 0). Kaplan-Meier survival plots were drawn using survminer (v0.4.9) and ggplot2 (v3.3.6).

#### DLBCL mutational and translocation status analysis by HERV cluster

Mutational and translocation status of DLBCL samples was obtained from the GDC Data Portal. The data was processed to retain only relevant mutations and structural variations. To facilitate downstream analysis, mutations were binarized into mutant (1) or wildtype (0) states for each gene or fusion event per sample. To assess the association between mutational status and cluster assignments, Fisher’s exact test was performed for each gene or fusion event across the identified HERV clusters.

### Quantification and statistical analyses

All analyses were performed in Bash, R (v4.0.2), and the BioConductor package manager (v1.30.19). Significance values for all DE analyses were calculated with the Wald test, with the Benjamini and Hochberg method for multiple testing correction. Comparisons between mean HERV and gene expression were conducted with the t-test, on normalized counts from DESeq2. Feature selection was performed using the multiple likelihood ratio test in DESeq2, the Boruta random forest algorithm, and the randomized LASSO regression. Enrichment of mutational signatures in HERV-based DLBCL clusters was calculated using Fisher’s exact test. Specific statistical details for all methods are outlined in detail under the respective method details sections and figure legends.

## Supplemental information

Document S1. Figures S1–S18 and Table S1

Table S2. Top 10 differentially expressed HERVs in each B-cell subtype in the B-AG dataset, related to Figure 1

Table S3. Top genes and HERVs from the B-AG dataset used to create B-cell-specific sets for HAGSEAS analysis, related to Figure 3

## References

[1] Lander E.S., Linton L.M., Birren B., Nusbaum C., Zody M.C., Baldwin J., Devon K., Dewar K., Doyle M., FitzHugh W. (2001). Initial sequencing and analysis of the human genome. Nature.

[2] Nurk S., Koren S., Rhie A., Rautiainen M., Bzikadze A.V., Mikheenko A., Vollger M.R., Altemose N., Uralsky L., Gershman A. (2022). The complete sequence of a human genome. Science.

[3] Bourque G., Burns K.H., Gehring M., Gorbunova V., Seluanov A., Hammell M., Imbeault M., Izsvák Z., Levin H.L., Macfarlan T.S. (2018). Ten things you should know about transposable elements. Genome Biol..

[4] Wells J.N., Feschotte C. (2020). A Field Guide to Eukaryotic Transposable Elements. Annu. Rev. Genet..

[5] Huang C.R.L., Burns K.H., Boeke J.D. (2012). Active transposition in genomes. Annu. Rev. Genet..

[6] de Parseval N., Heidmann T. (2005). Human endogenous retroviruses: from infectious elements to human genes. Cytogenet. Genome Res..

[7] Jern P., Coffin J.M. (2008). Effects of retroviruses on host genome function. Annu. Rev. Genet..

[8] She J., Du M., Xu Z., Jin Y., Li Y., Zhang D., Tao C., Chen J., Wang J., Yang E. (2022). The landscape of hervRNAs transcribed from human endogenous retroviruses across human body sites. Genome Biol..

[9] Fueyo R., Judd J., Feschotte C., Wysocka J. (2022). Roles of transposable elements in the regulation of mammalian transcription. Nat. Rev. Mol. Cell Biol..

[10] Chuong E.B., Elde N.C., Feschotte C. (2016). Regulatory evolution of innate immunity through co-option of endogenous retroviruses. Science.

[11] Dopkins N., Singh B., Michael S., O’Mara M.M., Marston J.L., Fei T., Bendall M.L., Nixon D.F. (2023). Endogenous Reverse Transcriptase Inhibition Attenuates TLR5-Mediated Inflammation. mBio.

[12] Lima-Junior D.S., Krishnamurthy S.R., Bouladoux N., Collins N., Han S.-J., Chen E.Y., Constantinides M.G., Link V.M., Lim A.I., Enamorado M. (2021). Endogenous retroviruses promote homeostatic and inflammatory responses to the microbiota. Cell.

[13] Mi S., Lee X., Li X., Veldman G.M., Finnerty H., Racie L., LaVallie E., Tang X.Y., Edouard P., Howes S. (2000). Syncytin is a captive retroviral envelope protein involved in human placental morphogenesis. Nature.

[14] Dupressoir A., Vernochet C., Bawa O., Harper F., Pierron G., Opolon P., Heidmann T. (2009). Syncytin-A knockout mice demonstrate the critical role in placentation of a fusogenic, endogenous retrovirus-derived, envelope gene. Proc. Natl. Acad. Sci. USA.

[15] Chen J., Foroozesh M., Qin Z. (2019). Transactivation of human endogenous retroviruses by tumor viruses and their functions in virus-associated malignancies. Oncogenesis.

[16] Kassiotis G. (2023). The Immunological Conundrum of Endogenous Retroelements. Annu. Rev. Immunol..

[17] Greenig M. (2019). HERVs, immunity, and autoimmunity: understanding the connection. PeerJ.

[18] Kassiotis G. (2014). Endogenous retroviruses and the development of cancer. J. Immunol..

[19] Kassiotis G., Stoye J.P. (2016). Immune responses to endogenous retroelements: taking the bad with the good. Nat. Rev. Immunol..

[20] Weiss R.A. (2016). Human endogenous retroviruses: friend or foe?. APMIS.

[21] Payer L.M., Burns K.H. (2019). Transposable elements in human genetic disease. Nat. Rev. Genet..

[22] Gorbunova V., Seluanov A., Mita P., McKerrow W., Fenyö D., Boeke J.D., Linker S.B., Gage F.H., Kreiling J.A., Petrashen A.P. (2021). The role of retrotransposable elements in ageing and age-associated diseases. Nature.

[23] Mesin L., Ersching J., Victora G.D. (2016). Germinal Center B Cell Dynamics. Immunity.

[24] Suan D., Sundling C., Brink R. (2017). Plasma cell and memory B cell differentiation from the germinal center. Curr. Opin. Immunol..

[25] Basso K., Dalla-Favera R. (2015). Germinal centres and B cell lymphomagenesis. Nat. Rev. Immunol..

[26] Basso K. (2021). Biology of Germinal Center B Cells Relating to Lymphomagenesis. Hemasphere.

[27] Lock F.E., Rebollo R., Miceli-Royer K., Gagnier L., Kuah S., Babaian A., Sistiaga-Poveda M., Lai C.B., Nemirovsky O., Serrano I. (2014). Distinct isoform of FABP7 revealed by screening for retroelement-activated genes in diffuse large B-cell lymphoma. Proc. Natl. Acad. Sci. USA.

[28] Sutkowski N., Conrad B., Thorley-Lawson D.A., Huber B.T. (2001). Epstein-Barr Virus Transactivates the Human Endogenous Retrovirus HERV-K18 that Encodes a Superantigen. Immunity.

[29] Ye X., Wang L., Nie M., Wang Y., Dong S., Ren W., Li G., Li Z.-M., Wu K., Pan-Hammarström Q. (2022). A single-cell atlas of diffuse large B cell lymphoma. Cell Rep..

[30] Bhardwaj N., Maldarelli F., Mellors J., Coffin J.M. (2014). HIV-1 infection leads to increased transcription of human endogenous retrovirus HERV-K (HML-2) proviruses in vivo but not to increased virion production. J. Virol..

[31] Contreras-Galindo R., López P., Vélez R., Yamamura Y. (2007). HIV-1 Infection Increases the Expression of Human Endogenous Retroviruses Type K (HERV-K) in Vitro. AIDS Res. Hum. Retroviruses.

[32] Srinivasachar Badarinarayan S., Shcherbakova I., Langer S., Koepke L., Preising A., Hotter D., Kirchhoff F., Sparrer K.M.J., Schotta G., Sauter D. (2020). HIV-1 infection activates endogenous retroviral promoters regulating antiviral gene expression. Nucleic Acids Res..

[33] Frank J.A., Singh M., Cullen H.B., Kirou R.A., Benkaddour-Boumzaouad M., Cortes J.L., Garcia Pérez J., Coyne C.B., Feschotte C. (2022). Evolution and antiviral activity of a human protein of retroviral origin. Science.

[34] Garrison K.E., Jones R.B., Meiklejohn D.A., Anwar N., Ndhlovu L.C., Chapman J.M., Erickson A.L., Agrawal A., Spotts G., Hecht F.M. (2007). T cell responses to human endogenous retroviruses in HIV-1 infection. PLoS Pathog..

[35] Kyriakou E., Magiorkinis G. (2023). Interplay between endogenous and exogenous human retroviruses. Trends Microbiol..

[36] Bilajac E., Mahmutović L., Lundstrom K., Glamočlija U., Šutković J., Sezer A., Hromić-Jahjefendić A. (2022). Viral Agents as Potential Drivers of Diffuse Large B-Cell Lymphoma Tumorigenesis. Viruses.

[37] Babaian A., Romanish M.T., Gagnier L., Kuo L.Y., Karimi M.M., Steidl C., Mager D.L. (2016). Onco-exaptation of an endogenous retroviral LTR drives IRF5 expression in Hodgkin lymphoma. Oncogene.

[38] Richter J., John K., Staiger A.M., Rosenwald A., Kurz K., Michgehl U., Ott G., Franzenburg S., Kohler C., Finger J. (2022). Epstein–Barr virus status of sporadic Burkitt lymphoma is associated with patient age and mutational features. Br. J. Haematol..

[39] Hutcheson R.L., Chakravorty A., Sugden B. (2020). Burkitt Lymphomas Evolve to Escape Dependencies on Epstein-Barr Virus. Front. Cell. Infect. Microbiol..

[40] SoRelle E.D., Reinoso-Vizcaino N.M., Dai J., Barry A.P., Chan C., Luftig M.A. (2023). Epstein-Barr virus evades restrictive host chromatin closure by subverting B cell activation and germinal center regulatory loci. Cell Rep..

[41] Leung A., Trac C., Kato H., Costello K.R., Chen Z., Natarajan R., Schones D.E. (2018). LTRs activated by Epstein-Barr virus–induced transformation of B cells alter the transcriptome. Genome Res..

[42] Ren W., Ye X., Su H., Li W., Liu D., Pirmoradian M., Wang X., Zhang B., Zhang Q., Chen L. (2018). Genetic landscape of hepatitis B virus-associated diffuse large B-cell lymphoma. Blood.

[43] Ren W., Wang X., Yang M., Wan H., Li X., Ye X., Meng B., Li W., Yu J., Lei M. (2022). Distinct clinical and genetic features of hepatitis B virus-associated follicular lymphoma in Chinese patients. Blood Adv..

[44] Holmes A.B., Corinaldesi C., Shen Q., Kumar R., Compagno N., Wang Z., Nitzan M., Grunstein E., Pasqualucci L., Dalla-Favera R., Basso K. (2020). Single-cell analysis of germinal-center B cells informs on lymphoma cell of origin and outcome. J. Exp. Med..

[45] Schmitz R., Wright G.W., Huang D.W., Johnson C.A., Phelan J.D., Wang J.Q., Roulland S., Kasbekar M., Young R.M., Shaffer A.L. (2018). Genetics and pathogenesis of diffuse large B-Cell lymphoma. N. Engl. J. Med..

[46] Roberto M.P., Varano G., Vinas-Castells R., Holmes A.B., Kumar R., Pasqualucci L., Farinha P., Scott D.W., Dominguez-Sola D. (2021). Mutations in the transcription factor FOXO1 mimic positive selection signals to promote germinal center B cell expansion and lymphomagenesis. Immunity.

[47] Wright G.W., Huang D.W., Phelan J.D., Coulibaly Z.A., Roulland S., Young R.M., Wang J.Q., Schmitz R., Morin R.D., Tang J. (2020). A Probabilistic Classification Tool for Genetic Subtypes of Diffuse Large B Cell Lymphoma with Therapeutic Implications. Cancer Cell.

[48] Steen C.B., Luca B.A., Esfahani M.S., Azizi A., Sworder B.J., Nabet B.Y., Kurtz D.M., Liu C.L., Khameneh F., Advani R.H. (2021). The landscape of tumor cell states and ecosystems in diffuse large B cell lymphoma. Cancer Cell.

[49] Agirre X., Meydan C., Jiang Y., Garate L., Doane A.S., Li Z., Verma A., Paiva B., Martín-Subero J.I., Elemento O. (2019). Long non-coding RNAs discriminate the stages and gene regulatory states of human humoral immune response. Nat. Commun..

[50] Bendall M.L., de Mulder M., Iñiguez L.P., Lecanda-Sánchez A., Pérez-Losada M., Ostrowski M.A., Jones R.B., Mulder L.C.F., Reyes-Terán G., Crandall K.A. (2019). Telescope: Characterization of the retrotranscriptome by accurate estimation of transposable element expression. PLoS Comput. Biol..

[51] Steiner M.C., Marston J.L., Iñiguez L.P., Bendall M.L., Chiappinelli K.B., Nixon D.F., Crandall K.A. (2021). Locus-Specific Characterization of Human Endogenous Retrovirus Expression in Prostate, Breast, and Colon Cancers. Cancer Res..

[52] Burn A., Roy F., Freeman M., Coffin J.M. (2022). Widespread expression of the ancient HERV-K (HML-2) provirus group in normal human tissues. PLoS Biol..

[53] He J., Babarinde I.A., Sun L., Xu S., Chen R., Shi J., Wei Y., Li Y., Ma G., Zhuang Q. (2021). Identifying transposable element expression dynamics and heterogeneity during development at the single-cell level with a processing pipeline scTE. Nat. Commun..

[54] Kursa M.B., Rudnicki W.R. (2010). Feature selection with the boruta package. J. Stat. Softw..

[55] Sill M., Hielscher T., Becker N., Zucknick M. (2014). c060: Extended Inference with Lasso and Elastic-Net Regularized Cox and Generalized Linear Models. J. Stat. Softw..

[56] Korotkevich G., Sukhov V., Sergushichev A. (2016). Fast gene set enrichment analysis. bioRxiv.

[57] Wilkerson M.D., Hayes D.N. (2010). ConsensusClusterPlus: A class discovery tool with confidence assessments and item tracking. Bioinformatics.

[58] Love M.I., Huber W., Anders S. (2014). Moderated estimation of fold change and dispersion for RNA-seq data with DESeq2. Genome Biol..

[59] Subramanian A., Tamayo P., Mootha V.K., Mukherjee S., Ebert B.L., Gillette M.A., Paulovich A., Pomeroy S.L., Golub T.R., Lander E.S., Mesirov J.P. (2005). Gene set enrichment analysis: A knowledge-based approach for interpreting genome-wide expression profiles. Proc. Natl. Acad. Sci. USA.

[60] Liberzon A., Birger C., Thorvaldsdóttir H., Ghandi M., Mesirov J.P., Tamayo P. (2015). The Molecular Signatures Database (MSigDB) hallmark gene set collection. Cell Syst..

[61] Rosenwald A., Wright G., Chan W.C., Connors J.M., Campo E., Fisher R.I., Gascoyne R.D., Muller-Hermelink H.K., Smeland E.B., Giltnane J.M. (2002). The use of molecular profiling to predict survival after chemotherapy for diffuse large-B-cell lymphoma. N. Engl. J. Med..

[62] Schulze A., Oshi M., Endo I., Takabe K. (2020). MYC Targets Scores Are Associated with Cancer Aggressiveness and Poor Survival in ER-Positive Primary and Metastatic Breast Cancer. Int. J. Mol. Sci..

[63] Dewannieux M., Blaise S., Heidmann T. (2005). Identification of a Functional Envelope Protein from the HERV-K Family of Human Endogenous Retroviruses. J. Virol..

[64] Ruggieri A., Maldener E., Sauter M., Mueller-Lantzsch N., Meese E., Fackler O.T., Mayer J. (2009). Human endogenous retrovirus HERV-K(HML-2) encodes a stable signal peptide with biological properties distinct from Rec. Retrovirology.

[65] Huang G., Li Z., Wan X., Wang Y., Dong J. (2013). Human endogenous retroviral K element encodes fusogenic activity in melanoma cells. J. Carcinog..

[66] Bhardwaj N., Coffin J.M. (2014). Endogenous Retroviruses and Human Cancer: Is There Anything to the Rumors?. Cell Host Microbe.

[67] Treangen T.J., Salzberg S.L. (2011). Repetitive DNA and next-generation sequencing: computational challenges and solutions. Nat. Rev. Genet..

[68] Dopkins N., Nixon D.F. (2024). Activation of human endogenous retroviruses and its physiological consequences. Nat. Rev. Mol. Cell Biol..

[69] Schwarz R., Koch P., Wilbrandt J., Hoffmann S. (2022). Locus-specific expression analysis of transposable elements. Brief. Bioinform..

[70] Lanciano S., Cristofari G. (2020). Measuring and interpreting transposable element expression. Nat. Rev. Genet..

[71] Yang W.R., Ardeljan D., Pacyna C.N., Payer L.M., Burns K.H. (2019). SQuIRE reveals locus-specific regulation of interspersed repeat expression. Nucleic Acids Res..

[72] Tokuyama M., Kong Y., Song E., Jayewickreme T., Kang I., Iwasaki A. (2018). ERVmap analysis reveals genome-wide transcription of human endogenous retroviruses. Proc. Natl. Acad. Sci. USA.

[73] Köster J., Rahmann S. (2012). Snakemake—a scalable bioinformatics workflow engine. Bioinformatics.

[74] Chapuy B., Stewart C., Dunford A.J., Kim J., Kamburov A., Redd R.A., Lawrence M.S., Roemer M.G.M., Li A.J., Ziepert M. (2018). Molecular subtypes of diffuse large B cell lymphoma are associated with distinct pathogenic mechanisms and outcomes. Nat. Med..

[75] Weinstein J.N., Collisson E.A., Mills G.B., Shaw K.R.M., Ozenberger B.A., Ellrott K., Shmulevich I., Sander C., Stuart J.M., Cancer Genome Atlas Research Network (2013). The Cancer Genome Atlas Pan-Cancer analysis project. Nat. Genet..

[76] Bendall M.L., Francis J.H., Shoushtari A.N., Nixon D.F. (2022). Specific human endogenous retroviruses predict metastatic potential in uveal melanoma. JCI Insight.

[77] Kanehisa M., Sato Y., Kawashima M., Furumichi M., Tanabe M. (2016). KEGG as a reference resource for gene and protein annotation. Nucleic Acids Res..

