# Supplementary material for: Locus-specific human endogenous retroviruses reveal lymphoma subtypes
Source: iScience. 2025 Apr 28;28(6):112541. doi: 10.1016/j.isci.2025.112541 (PMC12141099; doi:10.1016/j.isci.2025.112541)
Supplement: Document S1. Figures S1–S18 and Table S1 [file mmc1.pdf]

iScience, Volume *28*

Supplemental information

Locus-specific human endogenous retroviruses reveal lymphoma subtypes

Bhavya Singh, Nicholas Dopkins, Tongyi Fei, Jez L. Marston, Stephanie Michael, Helena Reyes-Gopar, Gislaine Curty, Jonas J. Heymann, Amy Chadburn, Peter Martin, Fabio E. Leal, Ethel Cesarman, Douglas F. Nixon, and Matthew L. Bendall

Supplementary Figure Legends

Supplementary Figure 1: Unique and differentially expressed HERV loci in the B-HM dataset, Related to Figure 1. A. Upset plot of the number of unique and shared HERVs upregulated in each B cell type ($p < 0.001$, log2fold change > 1.5**).** **B.** Upset plot of the number of unique and shared HERVs downregulated in each B cell type ($p < 0.001$, log2fold change > 1.5). **C.** Volcano plot of differentially expressed HERVs in all cell types versus DZ, **D.** all versus LZ, **E.** all versus MB, and **F.** all versus NB.

Supplementary Figure 2: Unique and differentially expressed HERV loci in the B-AG dataset, Related to Figure 1. A. Upset plot of the number of unique and shared HERVs upregulated in each B cell type ($p < 0.001$, log2fold change > 1.5**).** **B.** Upset plot of the number of unique and shared HERVs downregulated in each B cell type ($p < 0.001$, log2fold change > 1.5). **C.** Volcano plot of differentially expressed HERVs in all cell types versus DZ, **D.** all versus LZ, **E.** all versus MB, and **F.** all versus NB.

Supplementary Figure 3: Plasmablasts and bone marrow plasma cells express distinct HERV profiles compared to GC B cells in the B-AG dataset, Related to Figure 1. A. Volcano plot of differentially expressed HERVs in all cell types versus BMPC**, B.** all versus PB**. C.** Heatmap of the top 75 upregulated genes and HERVs in PB ($p < 0.001$, log2fold change > 1.5), and **D.** BMPC.

Supplementary Figure 4: Key features differentiating B-AG B cell subsets based on feature selection with DESeq2 LRT, Boruta, and Lasso, Related to Figure 1. A. UpsetR plot displaying the number of features selected by DESeq2 lowest likelihood ratio (LTR), the random forest classification with the Boruta algorithm, and the randomized least absolute shrinkage and

selection operator (LASSO) regression, with 11 features being selected by all three methods. **B.** Rpart decision tree, displaying that HERVP71A_8q24.13 differentiates plasma cells (PB and BMPC) from the rest of the B cells. HERVL_2p12a differentiates DZ from the remaining cell types, while HUERSP2_6p22.3 differentiates LZ from MB and NB**. C.** Normalized counts plotted for the 11 HERV features differentiating the B cell subtypes: ERVLB4_14q23.3, HERVL_2p12a, HERVP71A_8q24.13, MER61_19p12c, HARLEQUIN_19p12b, HERVFRD_2p12a, PABLB_7q11.21, HERVL_1q23.3a, HERVP71A_15q24.2, HUERSP2_6p22.3, ERVLE_6p25.1b.

Supplementary Figure 5: Total % of reads assigned to TEs and HERVs by lymphoma type and sub-type, Related to Figure 2. A. Mean of the percentage (%) of reads assigned to TEs in BL, DLBCL, and FL, and **B.** their respective subtypes. **C.** Mean of the percentage (%) of reads assigned to HERVs in BL, DLBCL, and FL, and **D**. their respective subtypes.

Supplementary Figure 6: HERV upregulation and downregulation in lymphoma subtypes, Related to Figure 2. A. Upset plot of the number of unique and shared HERVs upregulated in each cancer sub-type, including ABC, GCB, and unclassified DLBCL, sporadic and endemic BL by EBV status, and follicular lymphoma. **B.** Upset plot of the number of unique and shared HERVs downregulated in each cancer sub-type. **C.** Relative abundance of HERV families per lymphoma sub-type. GCB-DLBCL contains the highest number of upregulated HERV loci.

Supplementary Figure 7: Upregulation of DZ-associated HERVs in BL compared to DLBCL and FL, Related to Figure 2. Four DZ-associated HERVs are significantly upregulated in BL compared to DLBCL and FL, as determined by a t-test to compare the means ($p < 0.05$). **A.** MER61_3q13.11, **B.** HML5_1q22**, C.** HERV3_14q32.33, **D.** HARLEQUIN_19p12b.

Supplementary Figure 8, Related to Figure 2: Upregulation of PB-associated HARLEQUIN_1q32.1 in ABC-DLBCL compared to other lymphoma subtypes. HARLEQUIN_1q32.1, which is **A.** associated with BMPC and PB, is significantly upregulated in **B.** ABC-DLBCL compared to GCB-DLBCL and unclassified-DLBCL and BL (t-test, $p < 0.005$).

Supplementary Figure 9: Unsupervised HERV-based classification of DLBCL samples compared to previous classifications, Related to Figure 4. A. Alluvial plot of 529 DLBCL samples, and their respective class calls for the COO classifications, DBL Hit status, scCOO group, Chapuy group, EcoTyper class, and Lymphgen class, compared to the HERV-based clusters. Transcriptomic signatures do not clearly segregate the samples based on previous classification, as observed in **B.** Gene-based PCA plot of 529 DLBCL samples, colored by COO classification, **C.** HERV-based PCA plot of 529 DLBCL samples, colored by COO classification, **D.** Gene-based PCA plot of 529 DLBCL samples, colored by EcoTyper classes, **E.** HERV-based PCA plot of 529 DLBCL samples, colored by EcoTyper classes, **F.** Gene-based PCA plot of 529 DLBCL samples, colored by LymphGen classifications, and **G.** HERV-based PCA plot of 529 DLBCL samples, colored by LymphGen classifications.

Supplementary Figure 10: Key features differentiating B-AG B cell subsets based on feature selection with DESeq2 LRT, Boruta, and Lasso, Related to Figure 4. **A.** UpsetR plot displaying the number of features selected by DESeq2 lowest likelihood ratio (LTR), the random forest classification with the Boruta algorithm, and the randomized least absolute shrinkage and selection operator (LASSO) regression, with 3 features being selected by all three methods, and 4 by both LASSO and Boruta. **B** Normalized counts plotted for the 4 HERV features differentiating the B cell subtypes: **B.** HML2_7p22.1, **C.** HERVH_16p13.2e , **D.** HERVW_2q23.3, and **E.** HERVH_7q11.23a. **F.** Rpart decision tree, displaying that HERVH_16p13.2e differentiates HC7 from the remaining clusters. HERVW_2q23.3

differentiates HC1 and HC2 from the remaining clusters, and then further differentiates HC2 from HC1, where its expression is the highest. HML2_7p22.1 separates HC4 and HC6 from HC3, HC4, and HC7, and then further differentiates within the clusters. HERVH_7q11.23a differentiates HC2 from HC3, HC4 from HC6, and HC7 from HC3 and HC5.

Supplementary Figure 11: ABC-like DLBCL clusters with unique HERV signatures, Related to Figure 4. HC1 and HC2 clusters contained the highest number of ABC-DLBCL samples. Top 75 differentially expressed genes and HERVs ($p < 0.001$, log2fold change > 1.5) in **A.** HC1, and **B.** HC2.

Supplementary Figure 12: GCB-like DLBCL clusters with unique HERV signatures, Related to Figure 4. HC3 and HC4 clusters contained the highest number of GCB-DLBCL samples. Top 75 differentially expressed genes and HERVs ($p < 0.001$, log2fold change > 1.5) in **A.** HC3, and **B.** HC4.

Supplementary Figure 13: PB-like and Post-GCB DLBCL clusters with unique HERV signatures, Related to Figure 4. Top 75 differentially expressed genes and HERVs ($p < 0.001$, log2fold change > 1.5) in **A.** The HC5 cluster, which was most associated with the PB cell-of-origin, and **B.** HC7 cluster, which was enriched in PB, BMPC, and MB**.**

Supplementary Figure 14: HERV upregulation and downregulation in BL HERV clusters and clinical subtypes, Related to Figure 5. A. Volcano plot of differentially expressed HERVs in BL-C1 vs BL-C2 ($p < 0.001$, log2fold change > 1.5), **B.** EBV- versus EBV+. **C.** Relative abundance of loci assigned to HERV families the HERV-driven BL-C1 and BL-C2 clusters, and **D.** Comparing between all EBV negative, EBV positive, Endemic, Endemic EBV negative, Endemic EBV positive, Sporadic, Sporadic EBV negative.

Supplementary Figure 15: Expression of selected BL features in other lymphoma subtypes, Related to Figure 5. Feature selection of differentially expressed HERVs in the two BL clusters found 4 HERVs sufficient to distinguish between BL-C1 and BL-C2. The same HERVs are also expressed in DLBCL and FL, but at different levels**. A.** ERVLE_2p25.3c is expressed most in Sporadic BL EBV negative**, B.** MER61_4p16.3 is expressed across lymphoma types, **C.** ERV316A3_2q21.2b has the highest expression in sporadic BL EBV negative, and **D.** ERVLE_5p13.2c is expressed in all lymphoma types, but with highest expression in BL.

Supplementary Figure 16: Top enriched MSigDB gene sets and pathways in DLBCL HERV clusters, Related to Figure 6. A. Enrichment of Gene Ontology Biological Processes pathways for the seven HERV clusters, showcasing distinct enrichment patterns for each cluster. The most enriched pathways for HC1 were chromosome organization, chromatin remodeling, positive regulation of RNA metabolic process, ncRNA processes, mRNA metabolic process, and cellular response to DNA damage stimulus. The pathways most enriched in HC2 were rRNA processing, RNA processing, ribosome biogenesis, ribonucleoprotein complex biogenesis, ncRNA processing, ncRNA metabolic process, along with DNA metabolic process and chromosome organization. The pathways most enriched in HC3 were cell motility, cell adhesion, locomotion, epithelium development, response to endogenous stimulus. The pathways most enriched in HC4 were small molecule metabolic process, peptide and organonitrogen compound biosynthetic process, generation of precursor metabolites and energy, cytoplasmic translation, and amide metabolic processes. HC5 had an overall enrichment of immune response signatures. HC6 and HC7 did not have any positive enrichment. **B.** Enrichment of BioCarta pathways for the seven HERV clusters. Similar to the

GO BP pathways, HC5 had the most striking enrichment of immune and inflammatory pathways.

Supplementary Figure 17: HERV-driven DLBCL subtypes have distinct biological properties and survival outcomes for unclassified DLBCL samples, Related to Figure 6. Survival plot of five DLBCL clusters containing unclassified cases. HC3 and HC6 contained only one unclassified case each and were thus omitted. HERV classes with the worst prognosis are HC2 and HC5, followed by HC1, HC4, and HC7.

Supplementary Figure 18: HERV-driven DLBCL subtypes and the DLBCL mutational landscape, Related to Figure 6. A. Heatmap of DLBCL samples and the corresponding mutations and translocations. **B.** Dotplot of mutational and translocation enrichment for each DLBCL HERV cluster. Dot size indicates the odd ratio, while red dots correspond to a significant enrichment ($p<0.05$, Fisher's Exact).

Supp Fig. 1

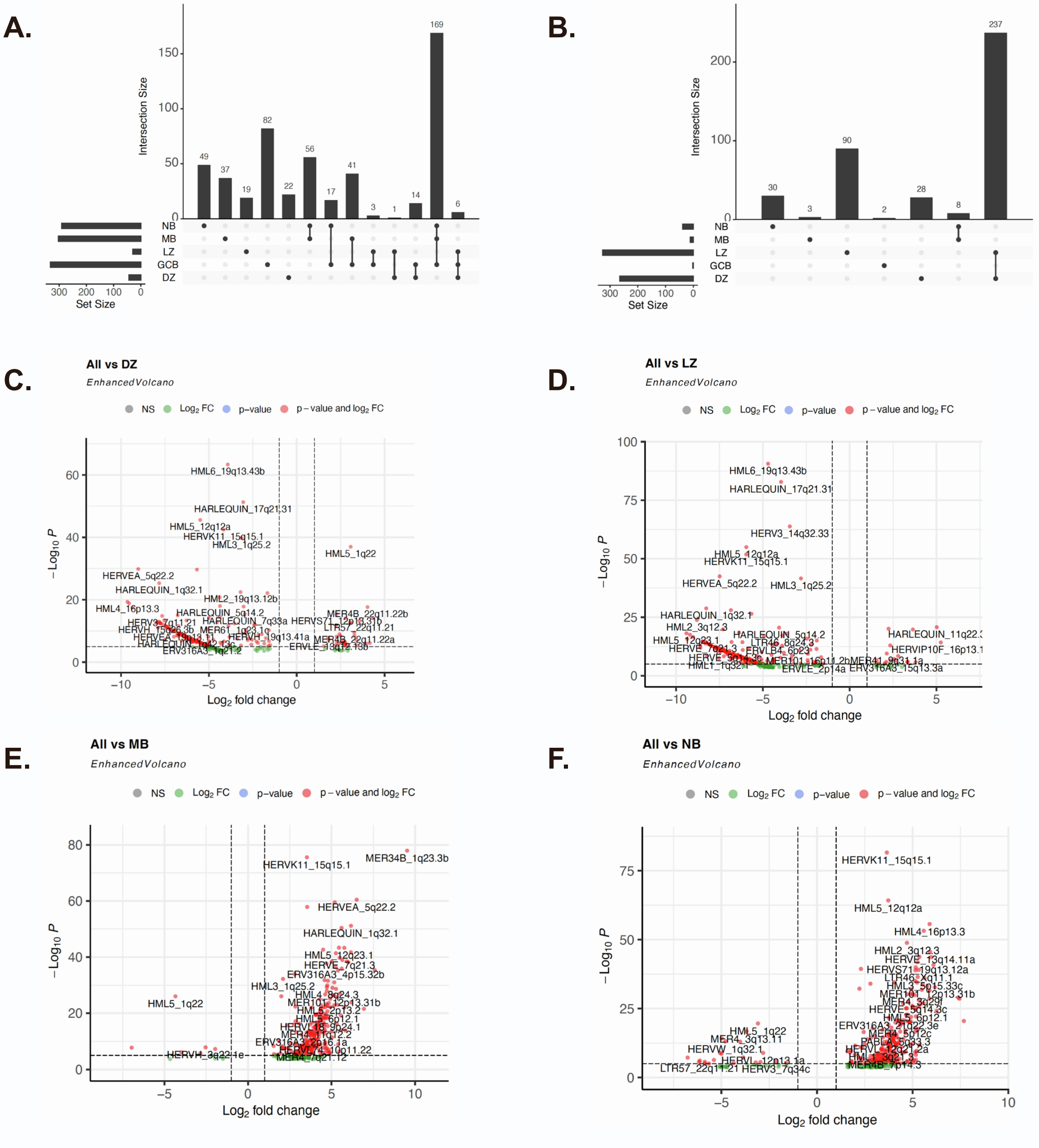
A.
B.
Intersection Size
Set Size
NB
MB
LZ
GCB
DZ
C.
All vs DZ
EnhancedVolcano
D.
All vs LZ
EnhancedVolcano
E.
All vs MB
EnhancedVolcano
F.
All vs NB
EnhancedVolcano
NS
p-value
p - value and log2 FC
Log2 fold change
HML6_19q13.43b
HARLEQUIN_17q21.31
HERVK11_15q15.1
HML5_1q22
MER34B_1q23.3b
HERVEA_5q22.2
HARLEQUIN_1q32.1
HML5_12q12a
HML4_16p13.3
HERV3_14q32.33
HML3_1q25.2

Supp Fig. 2

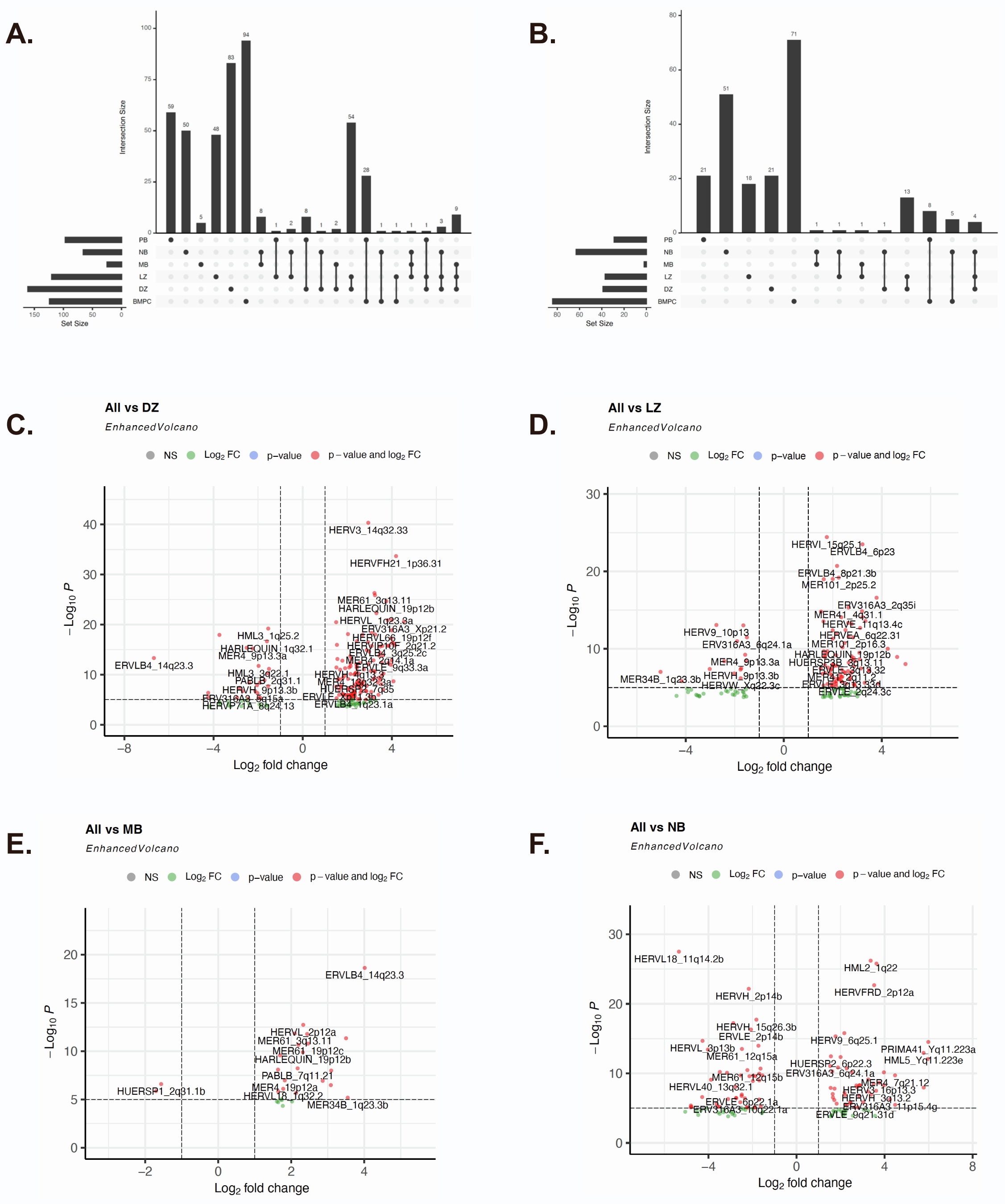
A.
Intersection Size
Set Size
PB
NB
MB
LZ
DZ
BMPC
B.
C.
All vs DZ
EnhancedVolcano
NS
p – value
p – value and log2 FC
HERV3_14q32.33
HERVFH21_1p36.31
MER61_3q13.11
HARLEQUIN_19p12b
HERVL_1q23.3a
ERV316A3_Xp21.2
HML3_1q25.2
HARLEQUIN_1q32.1
MER4_9p13.3a
ERVLB4_14q23.3
HML3_3q22.1
PABLB_2q31.1
HERVH_9p13.3b
ERVLB4_1q23.1a
Log2 fold change
D.
All vs LZ
HERVI_15q25.1
ERVLB4_6p23
ERVLB4_8p21.3b
MER101_2p25.2
ERV316A3_2q35i
MER41_4q31.1
HERVE_11q13.4c
HERVEA_6q22.31
HERV9_10p13
ERV316A3_6q24.1a
MER101_2p16.3
HARLEQUIN_19p12b
MER4_9p13.3a
MER34B_1q23.3b
HERVH_9p13.3b
HERVW_Xq22.3c
ERVLE_2q24.3c
E.
All vs MB
ERVLB4_14q23.3
HERVL_2p12a
MER61_3q13.11
MER61_19p12c
HARLEQUIN_19p12b
PABLB_7q11.21
HUERSP1_2q31.1b
MER4_19p12a
HERVL18_1q32.2
MER34B_1q23.3b
F.
All vs NB
HERVL18_11q14.2b
HML2_1q22
HERVFRD_2p12a
HERVH_2p14b
HERVH_15q26.3b
ERVLE_2p14b
HERV9_6q25.1
HERVL_3p13b
MER61_12q15a
PRIMA41_Yq11.223a
HML5_Yq11.223e
HUERSP2_6p22.3
ERV316A3_6q24.1a
MER61_12q15b
MER4_7q21.12
HERVL40_13q82.1
HERV3_16p13.3
HERVH_3q13.2
ERVLE_6p22.1a
ERV316A3_11p15.4g
ERV316A3_10q22.1a
ERVLE_9q21.31d

Supp Fig. 3

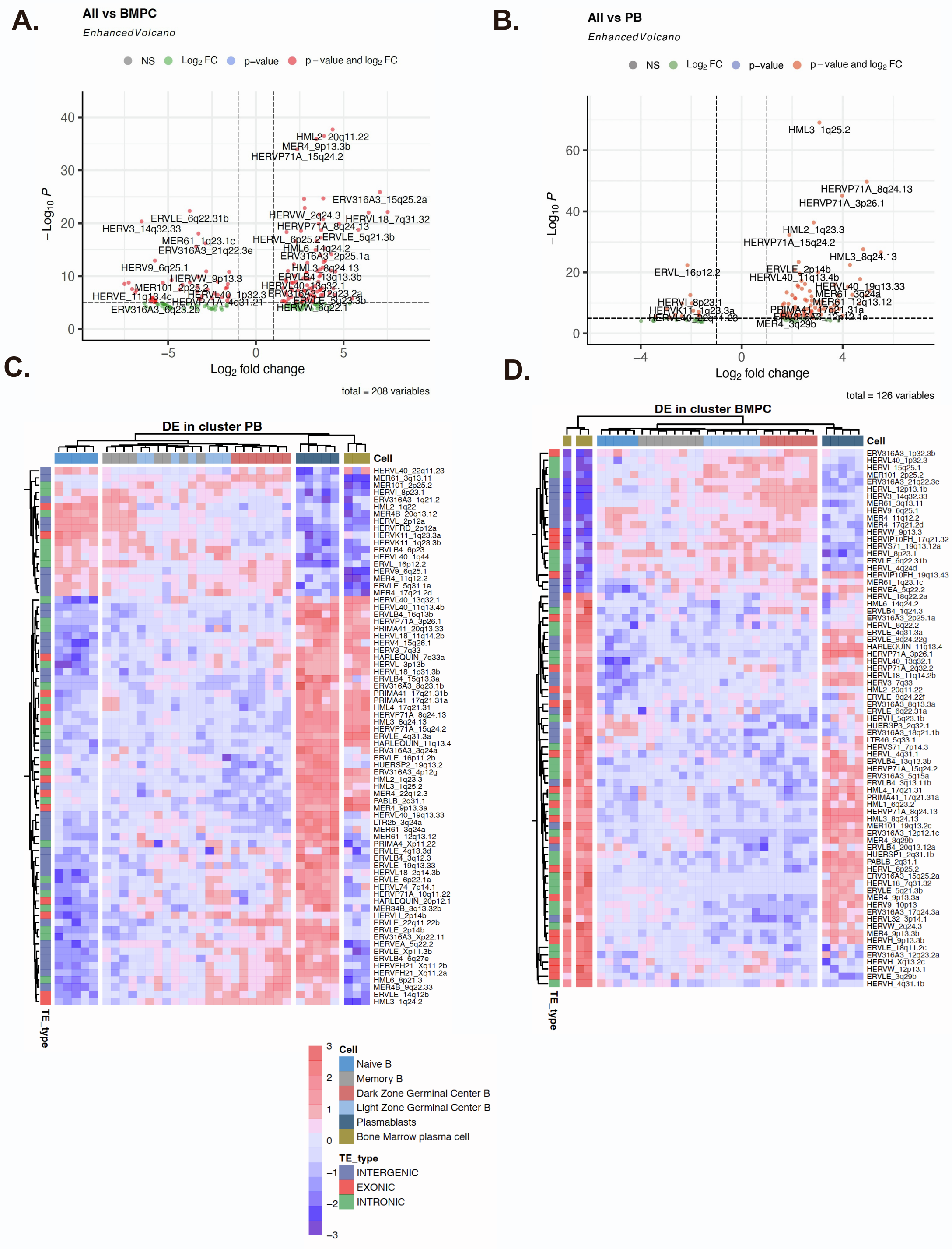

A.
All vs BMPC
EnhancedVolcano
NS
Log2 FC
p−value
p − value and log2 FC
−Log10 P
Log2 fold change
total = 208 variables
B.
All vs PB
EnhancedVolcano
NS
Log2 FC
p−value
p − value and log2 FC
−Log10 P
Log2 fold change
total = 126 variables
C.
DE in cluster PB
Cell
TE_type
D.
DE in cluster BMPC
Cell
TE_type
Cell
Naive B
Memory B
Dark Zone Germinal Center B
Light Zone Germinal Center B
Plasmablasts
Bone Marrow plasma cell
TE_type
INTERGENIC
EXONIC
INTRONIC

Supp Fig. 4

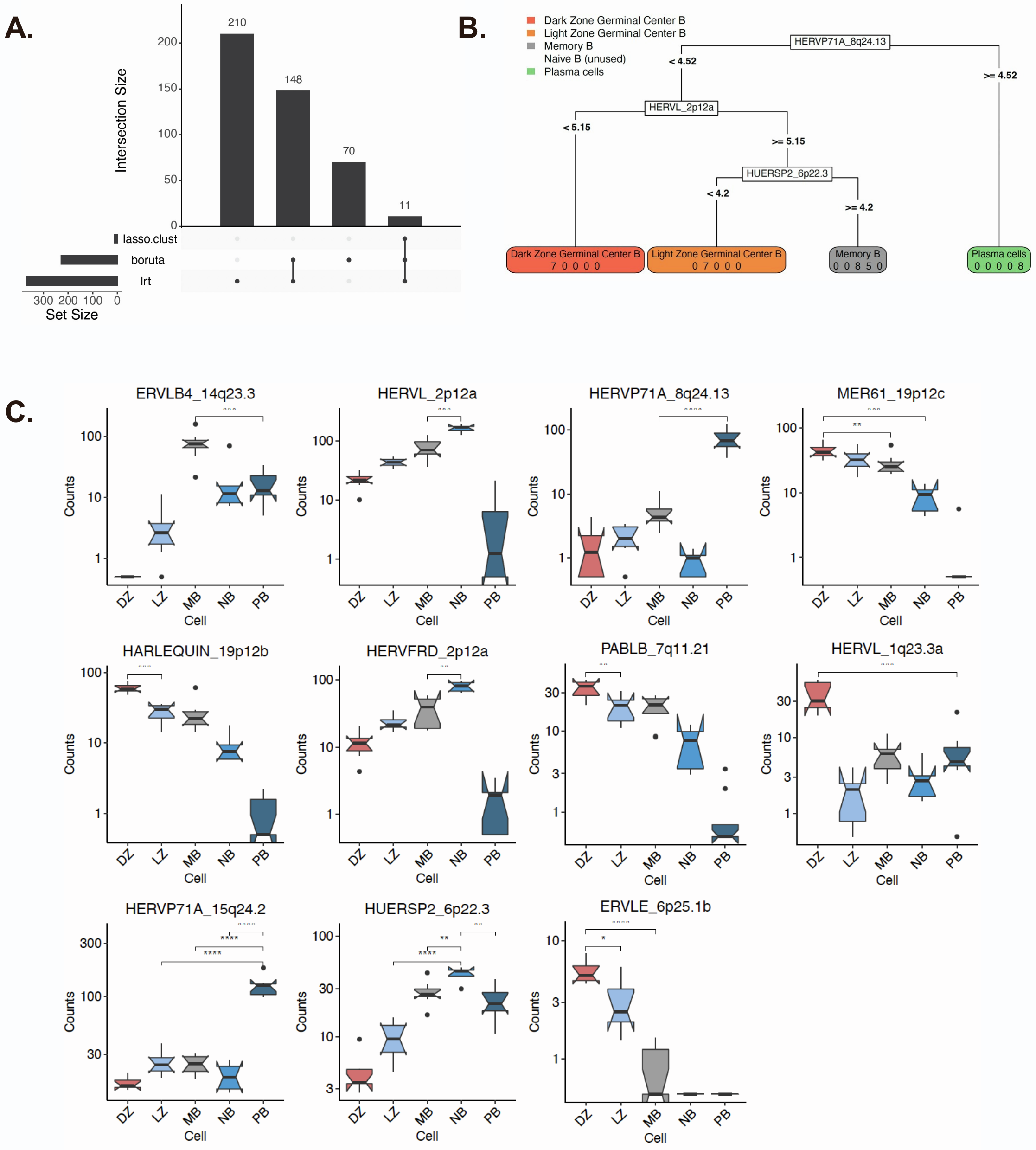
A.
Intersection Size
210
148
70
11
lasso.clust
boruta
lrt
Set Size
B.
Dark Zone Germinal Center B
Light Zone Germinal Center B
Memory B
Naive B (unused)
Plasma cells
HERVP71A_8q24.13
< 4.52
>= 4.52
HERVL_2p12a
< 5.15
>= 5.15
HUERSP2_6p22.3
< 4.2
>= 4.2
Dark Zone Germinal Center B 7 0 0 0 0
Light Zone Germinal Center B 0 7 0 0 0
Memory B 0 0 8 5 0
Plasma cells 0 0 0 0 8
C.
ERVLB4_14q23.3
HERVL_2p12a
HERVP71A_8q24.13
MER61_19p12c
HARLEQUIN_19p12b
HERVFRD_2p12a
PABLB_7q11.21
HERVL_1q23.3a
HERVP71A_15q24.2
HUERSP2_6p22.3
ERVLE_6p25.1b
Counts
Cell
DZ
LZ
MB
NB
PB

Supp Fig. 5

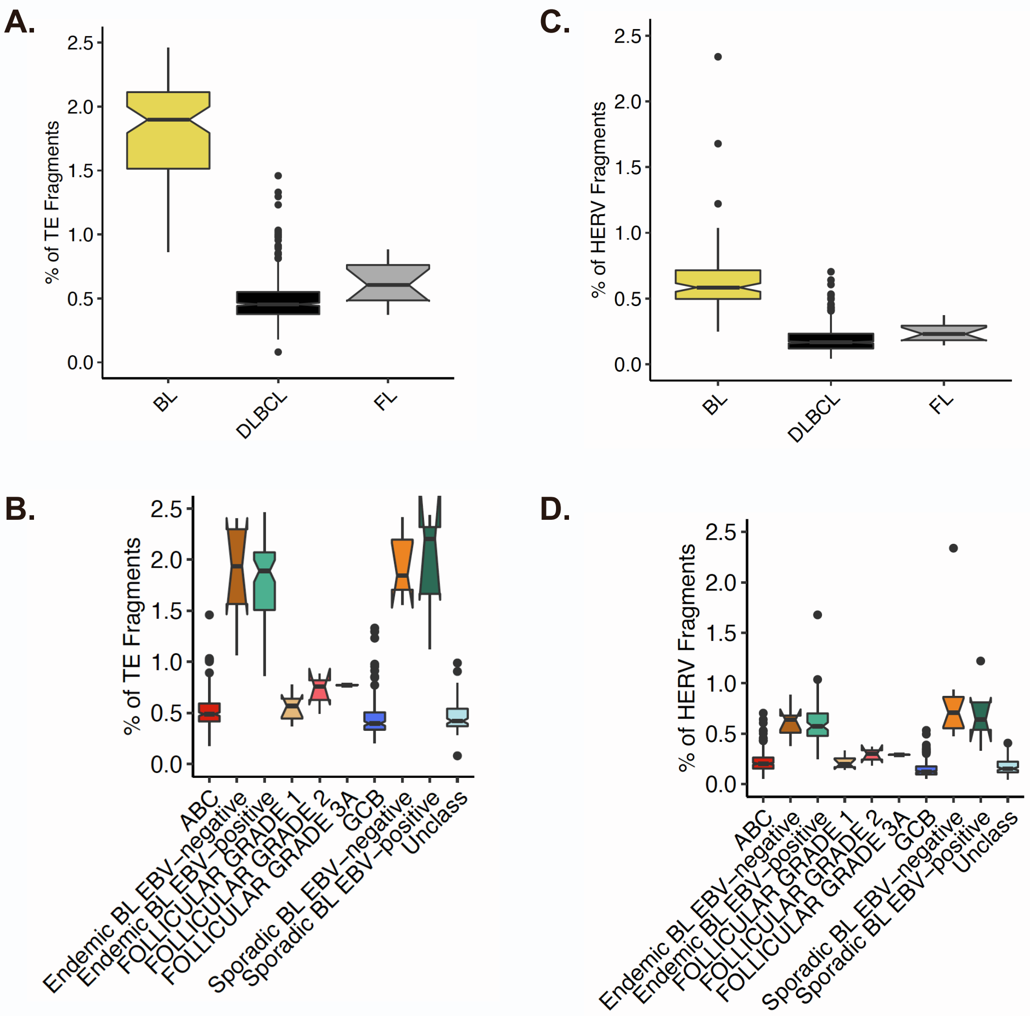

A.
2.5
2.0
1.5
1.0
0.5
0.0
% of TE Fragments
BL
DLBCL
FL
C.
% of HERV Fragments
B.
ABC
Endemic BL EBV−negative
Endemic BL EBV−positive
FOLLICULAR GRADE 1
FOLLICULAR GRADE 2
FOLLICULAR GRADE 3A
GCB
Sporadic BL EBV−negative
Sporadic BL EBV−positive
Unclass
D.

Supp Fig. 6

A.

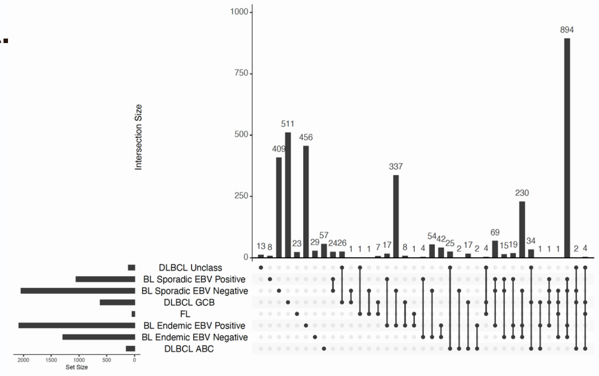

B.

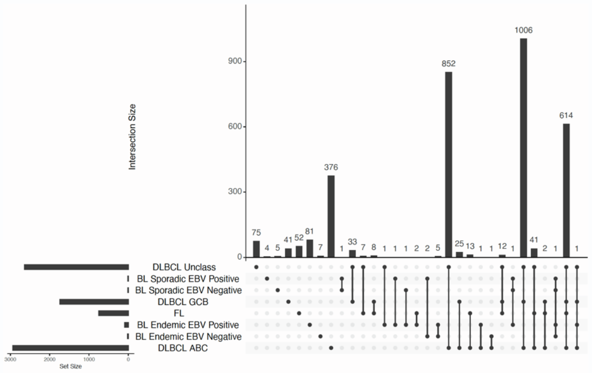

C.

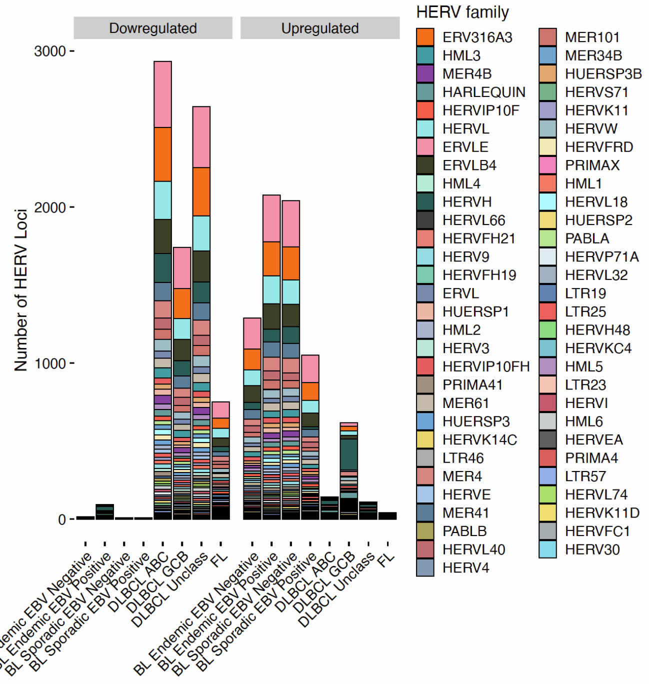

Supp Fig. 7

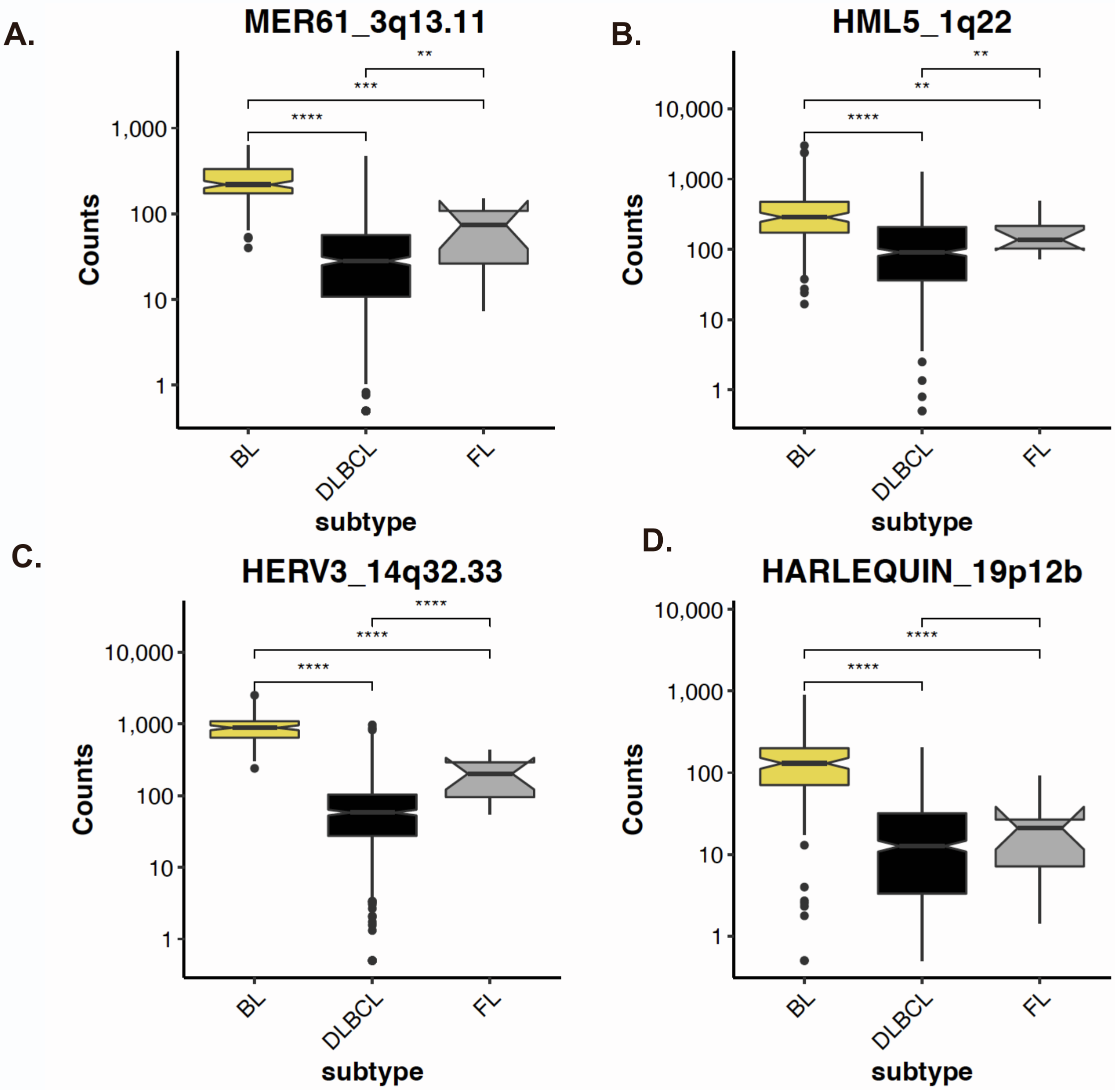
A.
MER61_3q13.11
**
***
****
1,000
100
10
1
Counts
BL
DLBCL
FL
subtype
B.
HML5_1q22
**
**
****
10,000
1,000
100
10
1
Counts
BL
DLBCL
FL
subtype
C.
HERV3_14q32.33
****
****
****
10,000
1,000
100
10
1
Counts
BL
DLBCL
FL
subtype
D.
HARLEQUIN_19p12b
****
****
10,000
1,000
100
10
1
Counts
BL
DLBCL
FL
subtype

Supp Fig. 8

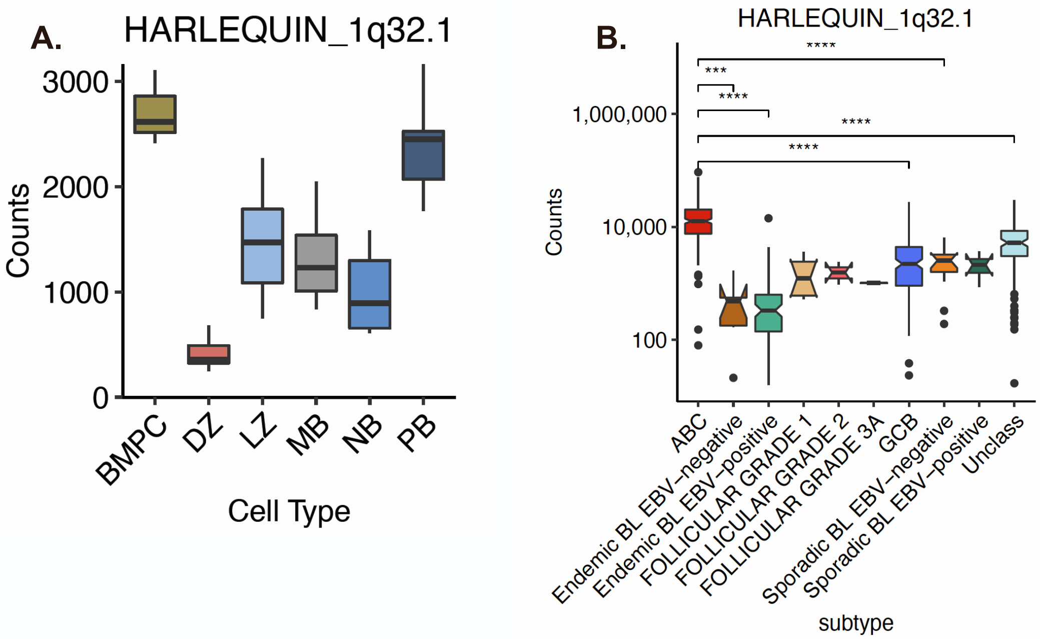
A.
HARLEQUIN_1q32.1
3000
2000
1000
0
Counts
BMPC
DZ
LZ
MB
NB
PB
Cell Type
B.
HARLEQUIN_1q32.1
1,000,000
10,000
100
Counts
****
***
****
****
****
ABC
Endemic BL EBV−negative
Endemic BL EBV−positive
FOLLICULAR GRADE 1
FOLLICULAR GRADE 2
FOLLICULAR GRADE 3A
GCB
Sporadic BL EBV−negative
Sporadic BL EBV−positive
Unclass
subtype

Supp Fig. 9

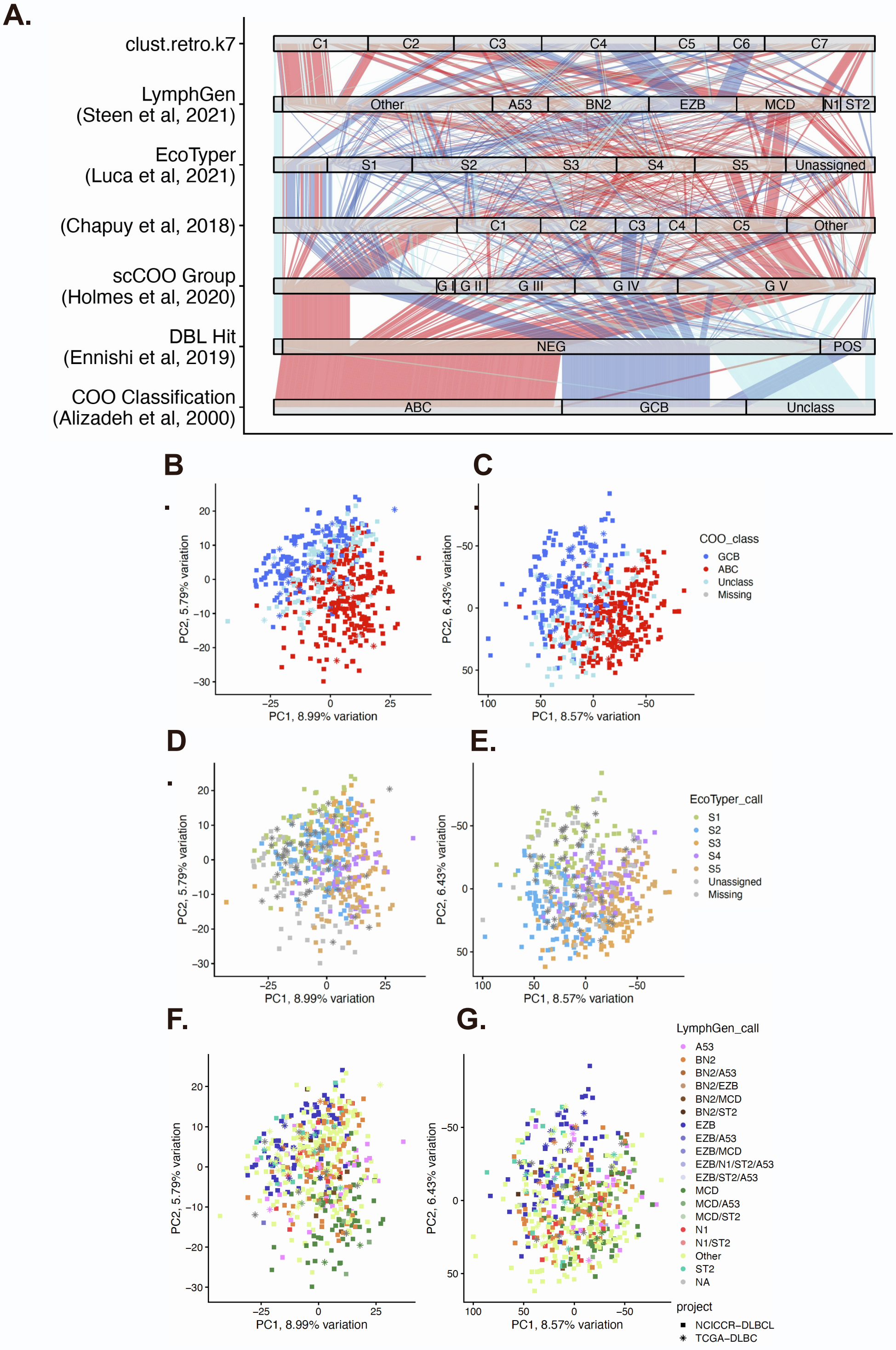
A.
clust.retro.k7
C1 C2 C3 C4 C5 C6 C7
LymphGen
(Steen et al, 2021)
Other A53 BN2 EZB MCD N1 ST2
EcoTyper
(Luca et al, 2021)
S1 S2 S3 S4 S5 Unassigned
(Chapuy et al, 2018)
C1 C2 C3 C4 C5 Other
scCOO Group
(Holmes et al, 2020)
G I G II G III G IV G V
DBL Hit
(Ennishi et al, 2019)
NEG POS
COO Classification
(Alizadeh et al, 2000)
ABC GCB Unclass
B
C
D
E.
F.
G.
PC1, 8.99% variation
PC2, 5.79% variation
PC1, 8.57% variation
PC2, 6.43% variation
COO_class
GCB
ABC
Unclass
Missing
EcoTyper_call
S1
S2
S3
S4
S5
Unassigned
Missing
LymphGen_call
A53
BN2
BN2/A53
BN2/EZB
BN2/MCD
BN2/ST2
EZB
EZB/A53
EZB/MCD
EZB/N1/ST2/A53
EZB/ST2/A53
MCD
MCD/A53
MCD/ST2
N1
N1/ST2
Other
ST2
NA
project
NCICCR−DLBCL
TCGA−DLBC

Supp Fig. 10

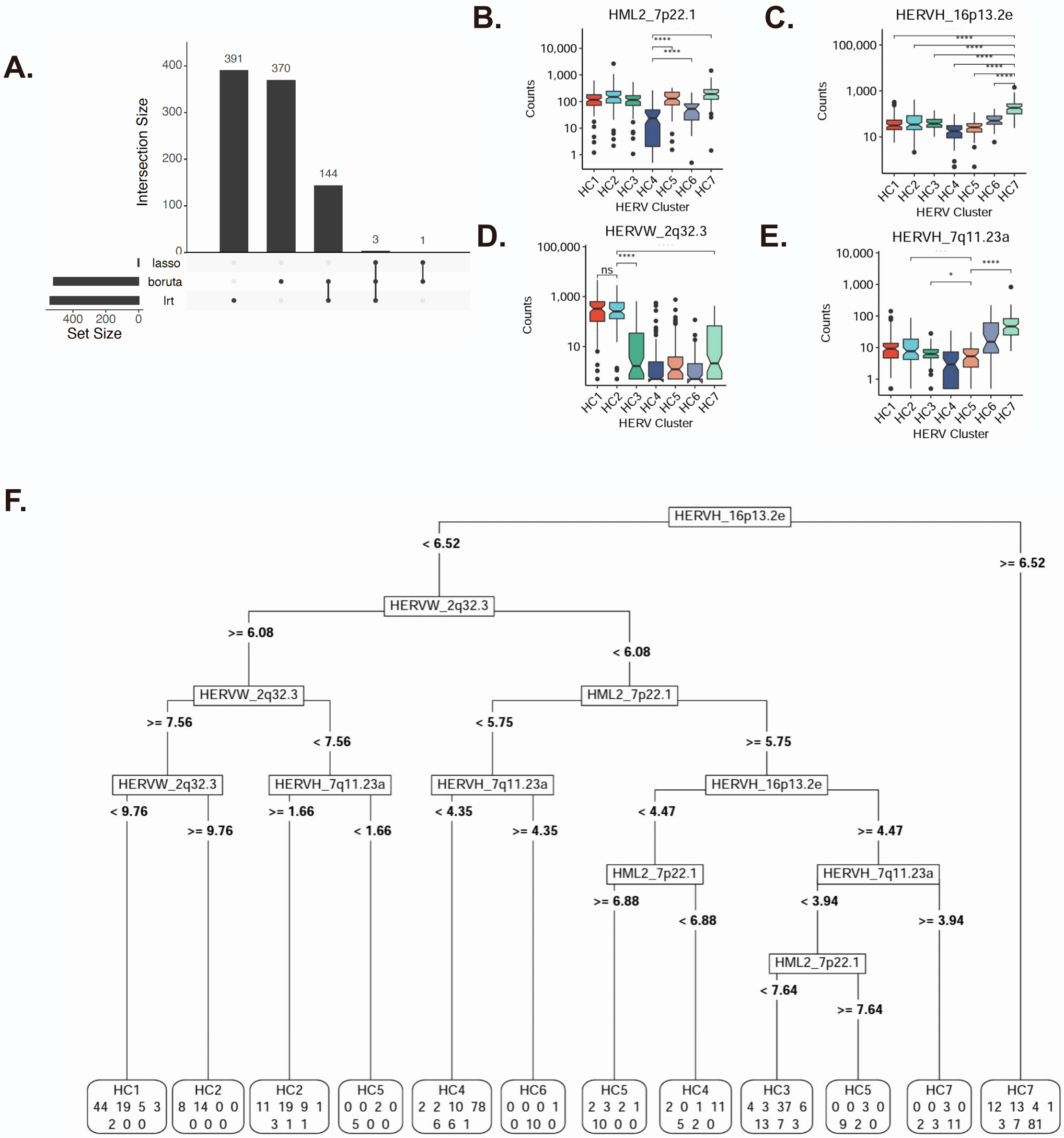
A.
Intersection Size
391
370
144
3
1
lasso
boruta
lrt
Set Size
B.
HML2_7p22.1
Counts
HERV Cluster
C.
HERVH_16p13.2e
D.
HERVW_2q32.3
ns
E.
HERVH_7q11.23a
F.
HERVH_16p13.2e
< 6.52
>= 6.52
HERVW_2q32.3
>= 6.08
< 6.08
HERVW_2q32.3
>= 7.56
< 7.56
HML2_7p22.1
< 5.75
>= 5.75
HERVW_2q32.3
< 9.76
>= 9.76
HERVH_7q11.23a
>= 1.66
< 1.66
HERVH_7q11.23a
< 4.35
>= 4.35
HERVH_16p13.2e
< 4.47
>= 4.47
HML2_7p22.1
>= 6.88
< 6.88
HERVH_7q11.23a
< 3.94
>= 3.94
HML2_7p22.1
< 7.64
>= 7.64
HC1 44 19 5 3 2 0 0
HC2 8 14 0 0 0 0 0
HC2 11 19 9 1 3 1 1
HC5 0 0 2 0 5 0 0
HC4 2 2 10 78 6 6 1
HC6 0 0 0 1 0 10 0
HC5 2 3 2 1 10 0 0
HC4 2 0 1 11 5 2 0
HC3 4 3 37 6 13 7 3
HC5 0 0 3 0 9 2 0
HC7 0 0 3 0 2 3 11
HC7 12 13 4 1 3 7 81

Supp Fig. 11

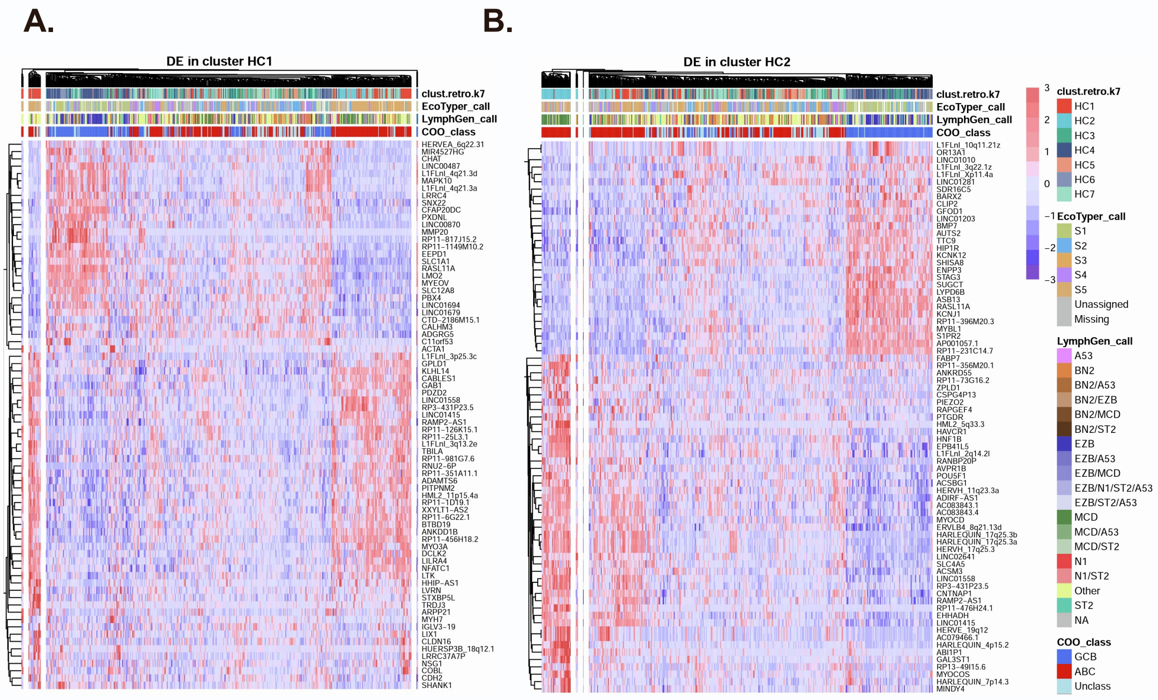
A.
B.
DE in cluster HC1
DE in cluster HC2
clust.retro.k7
EcoTyper_call
LymphGen_call
COO_class
3
2
1
0
−1
−2
−3
clust.retro.k7
HC1
HC2
HC3
HC4
HC5
HC6
HC7
EcoTyper_call
S1
S2
S3
S4
S5
Unassigned
Missing
LymphGen_call
A53
BN2
BN2/A53
BN2/EZB
BN2/MCD
BN2/ST2
EZB
EZB/A53
EZB/MCD
EZB/N1/ST2/A53
EZB/ST2/A53
MCD
MCD/A53
MCD/ST2
N1
N1/ST2
Other
ST2
NA
COO_class
GCB
ABC
Unclass

Supp Fig. 12

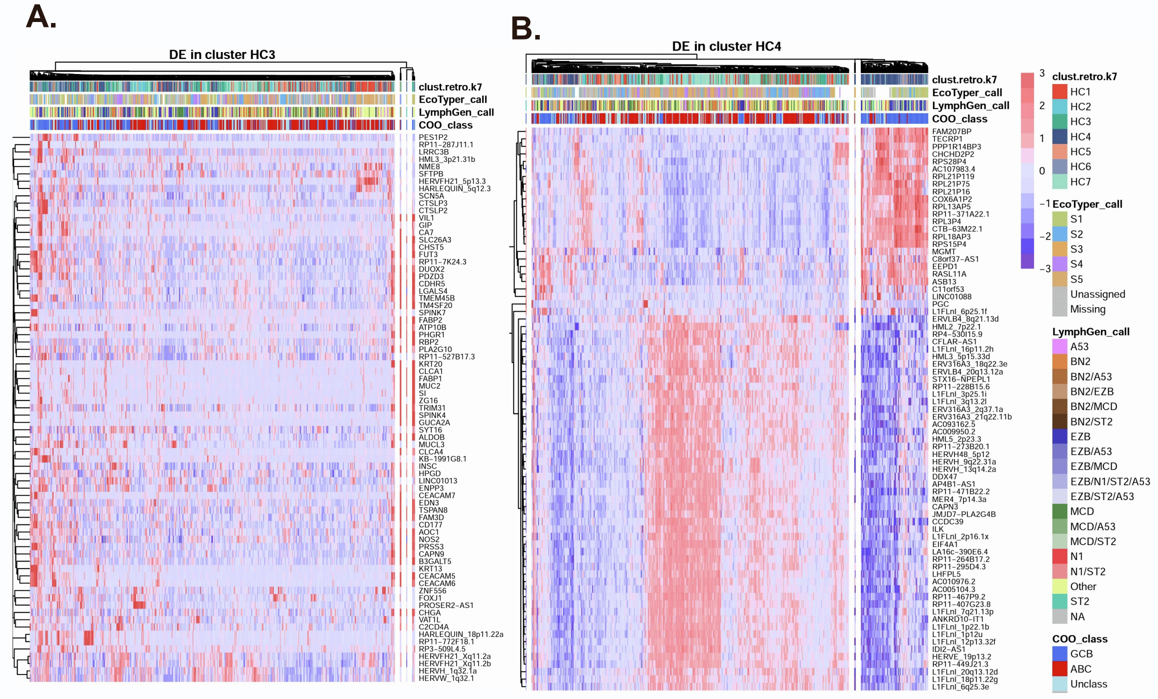
A.
DE in cluster HC3
clust.retro.k7
EcoTyper_call
LymphGen_call
COO_class
B.
DE in cluster HC4
clust.retro.k7
EcoTyper_call
LymphGen_call
COO_class
3
2
1
0
−1
−2
−3
clust.retro.k7
HC1
HC2
HC3
HC4
HC5
HC6
HC7
EcoTyper_call
S1
S2
S3
S4
S5
Unassigned
Missing
LymphGen_call
A53
BN2
BN2/A53
BN2/EZB
BN2/MCD
BN2/ST2
EZB
EZB/A53
EZB/MCD
EZB/N1/ST2/A53
EZB/ST2/A53
MCD
MCD/A53
MCD/ST2
N1
N1/ST2
Other
ST2
NA
COO_class
GCB
ABC
Unclass

Supp Fig. 13

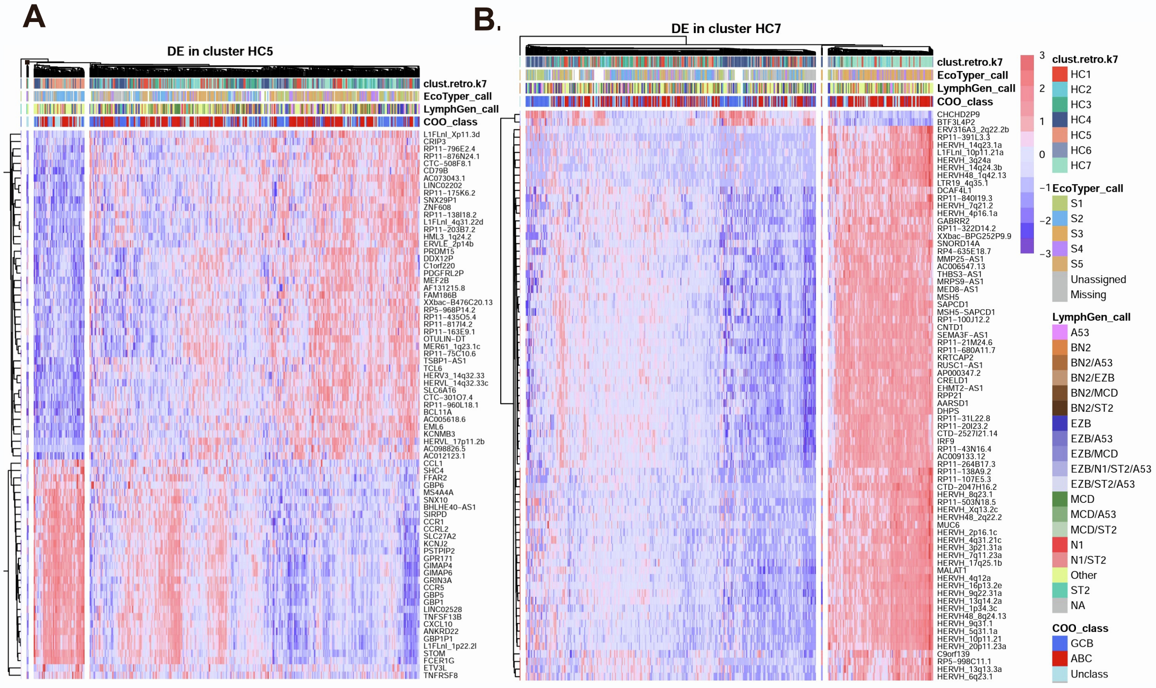

A
B.
DE in cluster HC5
DE in cluster HC7
clust.retro.k7
EcoTyper_call
LymphGen_call
COO_class
3
2
1
0
−1
−2
−3
clust.retro.k7
HC1
HC2
HC3
HC4
HC5
HC6
HC7
EcoTyper_call
S1
S2
S3
S4
S5
Unassigned
Missing
LymphGen_call
A53
BN2
BN2/A53
BN2/EZB
BN2/MCD
BN2/ST2
EZB
EZB/A53
EZB/MCD
EZB/N1/ST2/A53
EZB/ST2/A53
MCD
MCD/A53
MCD/ST2
N1
N1/ST2
Other
ST2
NA
COO_class
GCB
ABC
Unclass

Supp Fig. 14

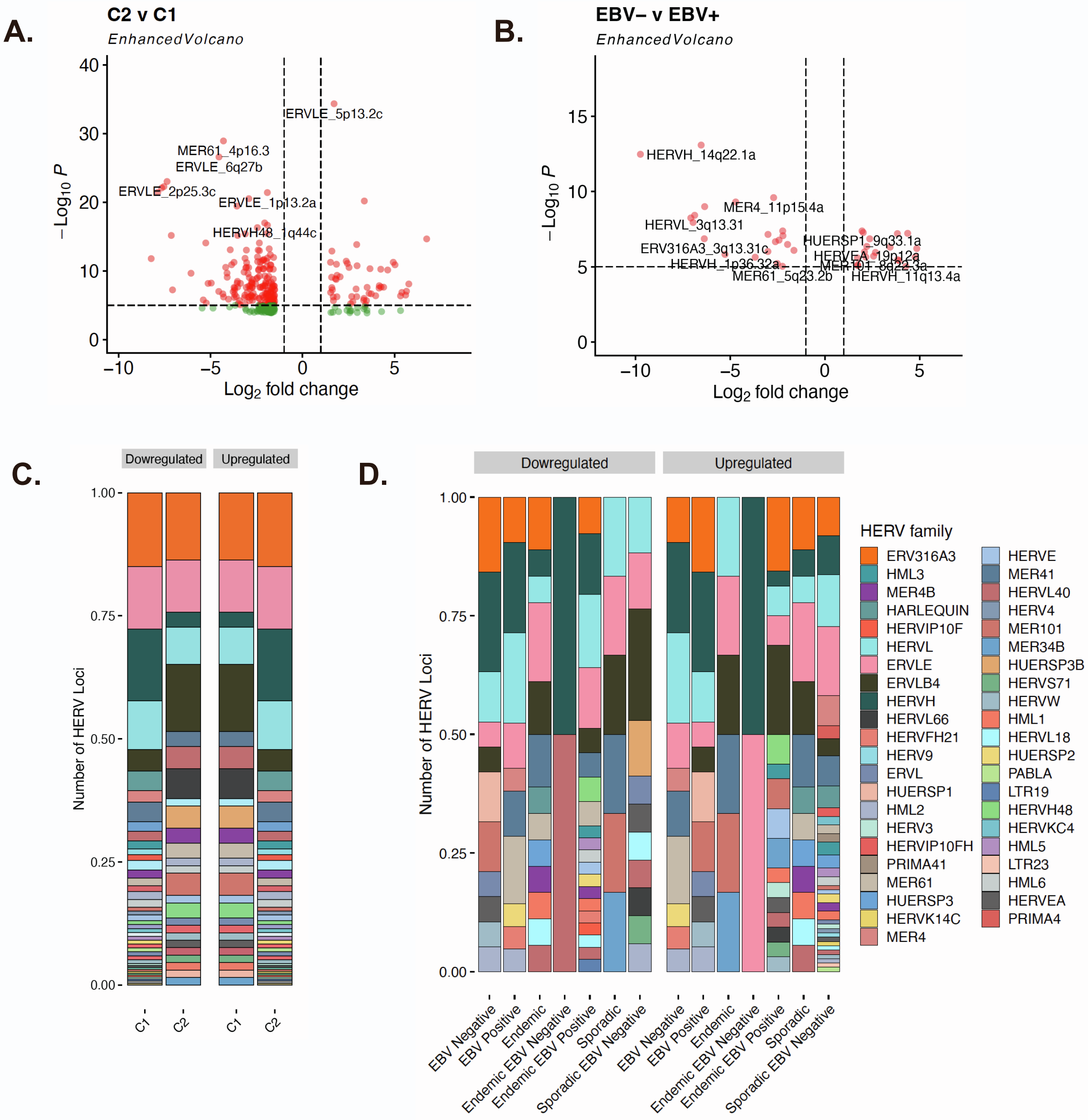
A.
C2 v C1
EnhancedVolcano
ERVLE_5p13.2c
MER61_4p16.3
ERVLE_6q27b
ERVLE_2p25.3c
ERVLE_1p13.2a
HERVH48_1q44c
−Log10 P
Log2 fold change
B.
EBV− v EBV+
EnhancedVolcano
HERVH_14q22.1a
MER4_11p15.4a
HERVL_3q13.31
ERV316A3_3q13.31c
HERVH_1p36.32a
MER61_5q23.2b
HUERSP1_9q33.1a
HERVEA_19p12a
MER101_8q22.3a
HERVH_11q13.4a
−Log10 P
Log2 fold change
C.
Dowregulated
Upregulated
Number of HERV Loci
C1
C2
D.
Dowregulated
Upregulated
Number of HERV Loci
EBV Negative
EBV Positive
Endemic
Endemic EBV Negative
Endemic EBV Positive
Sporadic
Sporadic EBV Negative
HERV family
ERV316A3
HML3
MER4B
HARLEQUIN
HERVIP10F
HERVL
ERVLE
ERVLB4
HERVH
HERVL66
HERVFH21
HERV9
ERVL
HUERSP1
HML2
HERV3
HERVIP10FH
PRIMA41
MER61
HUERSP3
HERVK14C
MER4
HERVE
MER41
HERVL40
HERV4
MER101
MER34B
HUERSP3B
HERVS71
HERVW
HML1
HERVL18
HUERSP2
PABLA
LTR19
HERVH48
HERVKC4
HML5
LTR23
HML6
HERVEA
PRIMA4

Supp Fig. 15

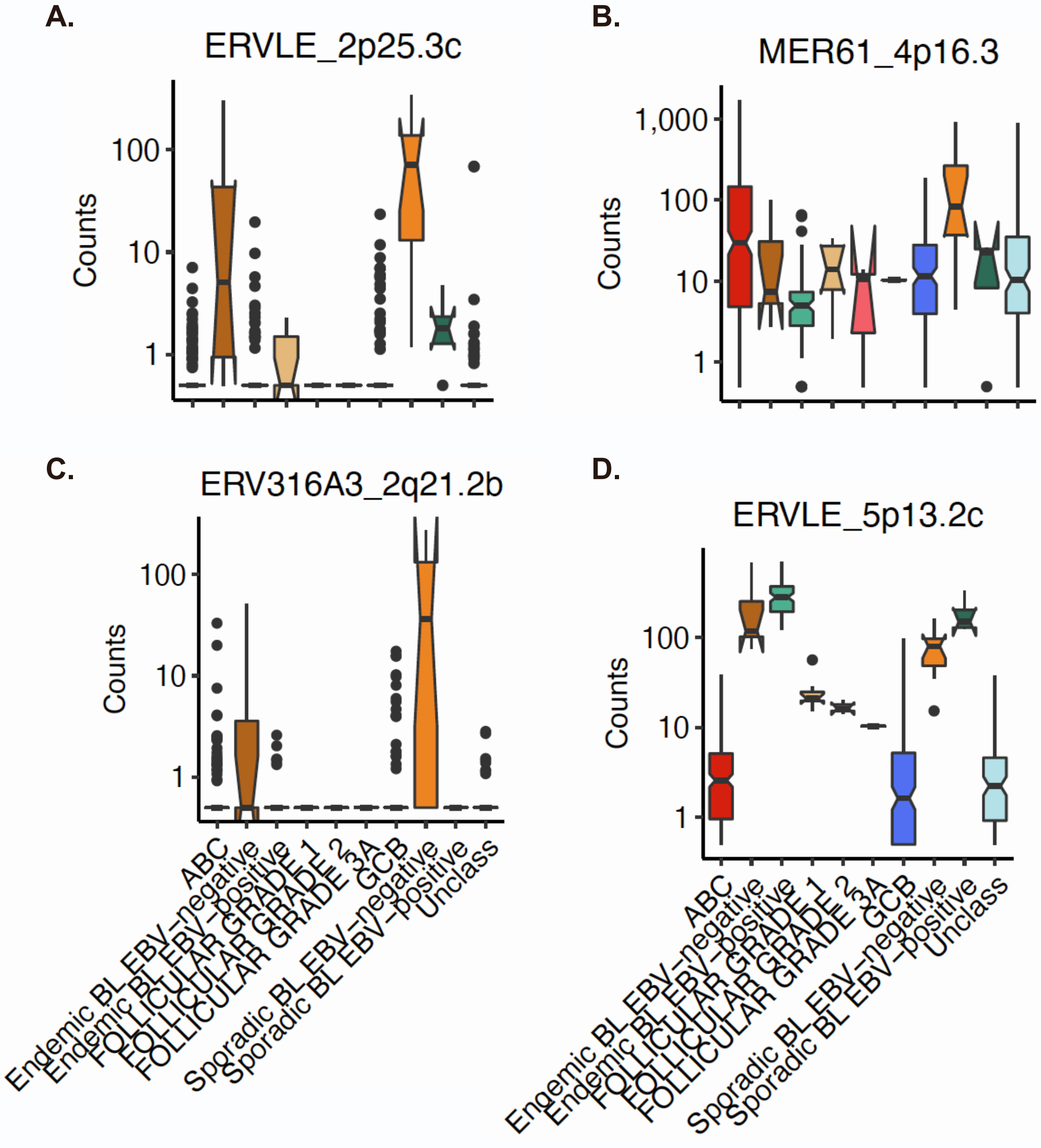

A.
ERVLE_2p25.3c
Counts
100
10
1
B.
MER61_4p16.3
Counts
1,000
100
10
1
C.
ERV316A3_2q21.2b
Counts
100
10
1
D.
ERVLE_5p13.2c
Counts
100
10
1
ABC
Endemic BL EBV−negative
Endemic BL EBV−positive
FOLLICULAR GRADE 1
FOLLICULAR GRADE 2
FOLLICULAR GRADE 3A
GCB
Sporadic BL EBV−negative
Sporadic BL EBV−positive
Unclass
ABC
Endemic BL EBV−negative
Endemic BL EBV−positive
FOLLICULAR GRADE 1
FOLLICULAR GRADE 2
FOLLICULAR GRADE 3A
GCB
Sporadic BL EBV−negative
Sporadic BL EBV−positive
Unclass

Supp Fig. 16

A.

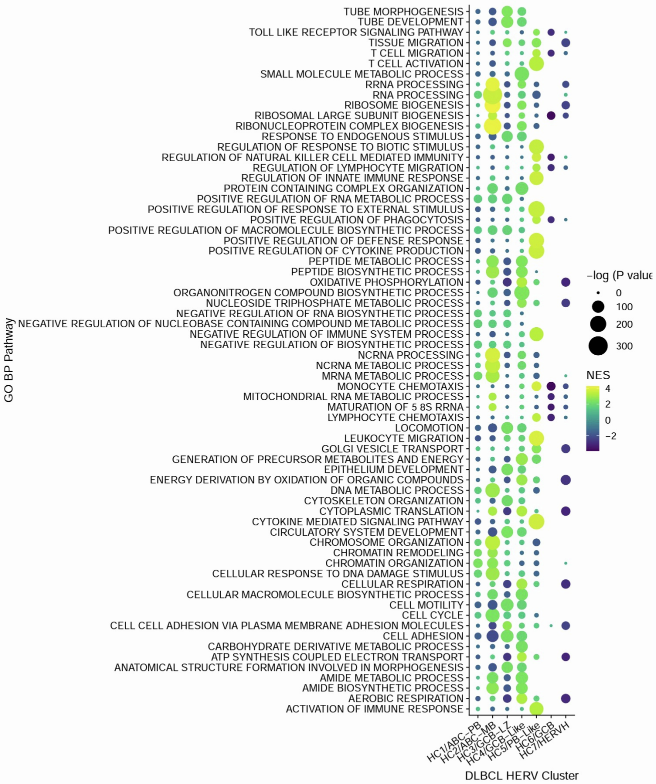

B.

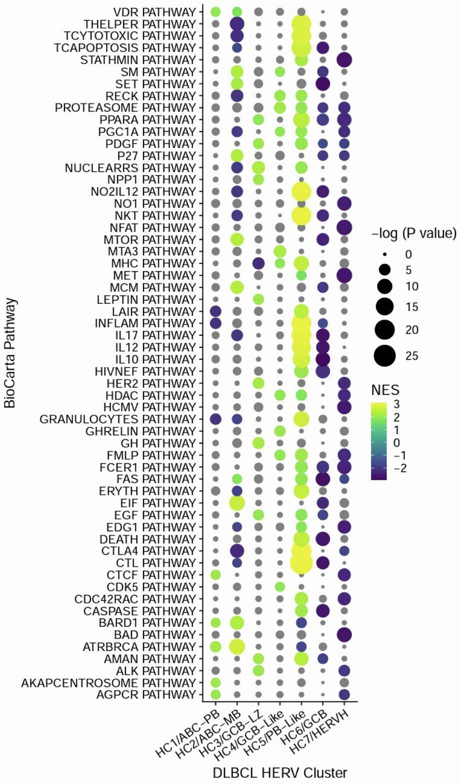

Supp Fig. 17

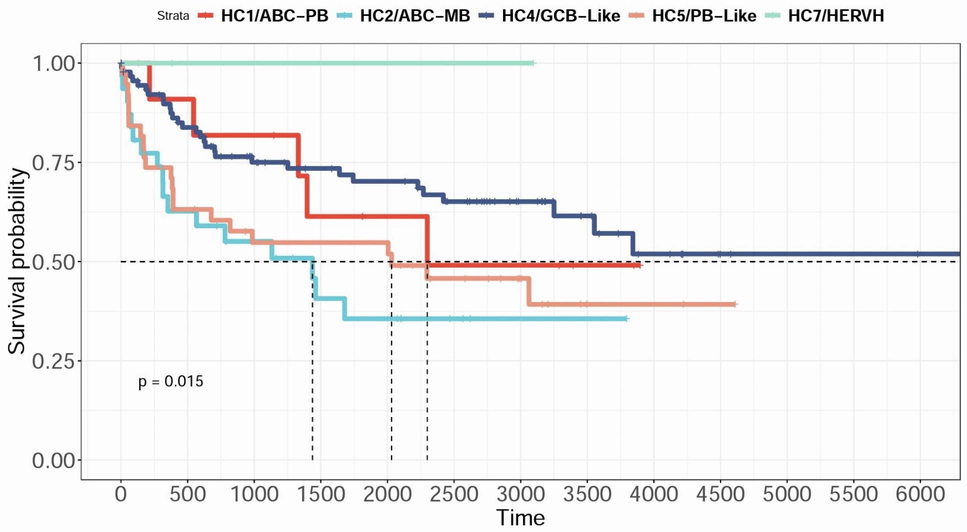

Strata
HC1/ABC-PB
HC2/ABC-MB
HC4/GCB-Like
HC5/PB-Like
HC7/HERVH
Survival probability
1.00
0.75
0.50
0.25
0.00
p = 0.015
0
500
1000
1500
2000
2500
3000
3500
4000
4500
5000
5500
6000
Time

Supp Fig. 18

A.

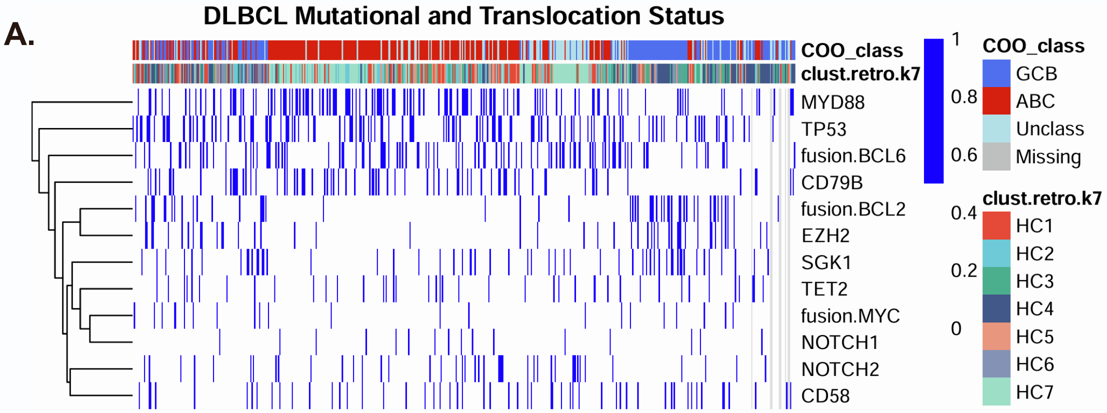

B.

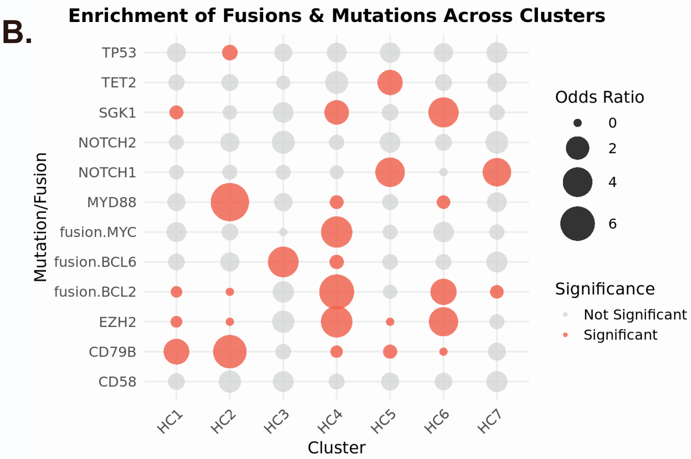

Supplementary Tables

	Number of Genes	Number of LINEs	Number of HERVs
**DLBLC (n=529)**	36,246	8,886	4,567
**BL (n=113)**	34,453	16,350	4,099
**FL (n=12)**	27,908	2,593	2,068
**B-AG (n=35)**	25,629	1,520	1,118
**B-HM (n=17)**	23,709	1,939	1,464

Supplementary Table 1: Number of coding genes, LINEs, and HERV loci remaining in each dataset after filtering, Related to Figure 1.

Supplementary Table 2: Top 10 differentially expressed HERVs in each B-cell subtype in the B-AG dataset, Related to Figure 1. Please refer to Excel-formatted table.

Supplementary Table 3: Top genes and HERVs from the B-AG dataset used to create B-cell-specific sets for HAGSEAS analysis, Related to Figure 3. Please refer to Excel-formatted table.
